# Pain Exposure and Brain Connectivity in Preterm Infants

**DOI:** 10.1001/jamanetworkopen.2024.2551

**Published:** 2024-03-15

**Authors:** Thiviya Selvanathan, Steven Ufkes, Ting Guo, Vann Chau, Helen M. Branson, George M. Ibrahim, Linh G. Ly, Edmond N. Kelly, Ruth E. Grunau, Steven P. Miller

**Affiliations:** 1 Department of Pediatrics, BC Children’s Hospital Research Institute and University of British Columbia, Vancouver, British Columbia, Canada; 2 Department of Pediatrics, The Hospital for Sick Children and University of Toronto, Toronto, Ontario, Canada; 3 Centre for Computational Medicine, The Hospital for Sick Children Research Institute, Toronto, Ontario, Canada; 4 Department of Diagnostic Imaging, The Hospital for Sick Children and Medical Imaging, University of Toronto, Toronto, Ontario, Canada; 5 Department of Surgery, The Hospital for Sick Children and University of Toronto, Toronto, Ontario, Canada; 6 Department of Pediatrics, Mount Sinai Hospital, Toronto, Ontario, Canada

## Abstract

**Importance:**

Early-life exposure to painful procedures has been associated with altered brain maturation and neurodevelopmental outcomes in preterm infants, although sex-specific differences are largely unknown.

**Objective:**

To examine sex-specific associations among early-life pain exposure, alterations in neonatal structural connectivity, and 18-month neurodevelopment in preterm infants.

**Design, Setting, and Participants:**

This prospective cohort study recruited 193 very preterm infants from April 1, 2015, to April 1, 2019, across 2 tertiary neonatal intensive care units in Toronto, Canada. Structural connectivity data were available for 150 infants; neurodevelopmental outcomes were available for 123 infants. Data were analyzed from January 1, 2022, to December 31, 2023.

**Exposure:**

Pain was quantified in the initial weeks after birth as the total number of invasive procedures.

**Main Outcome and Measure:**

Infants underwent early-life and/or term-equivalent-age magnetic resonance imaging with diffusion tensor imaging to quantify structural connectivity using graph theory measures and regional connection strength. Eighteen-month neurodevelopmental outcomes were assessed with the Bayley Scales of Infant and Toddler Development, Third Edition. Stratifying by sex, generalized estimating equations were used to assess whether pain exposure modified the maturation of structural connectivity using an interaction term (early-life pain exposure × postmenstrual age [PMA] at scan). Generalized estimating equations were used to assess associations between structural connectivity and neurodevelopmental outcomes, adjusting for extreme prematurity and maternal education.

**Results:**

A total of 150 infants (80 [53%] male; median [IQR] gestational age at birth, 27.1 [25.4-29.0] weeks) with structural connectivity data were analyzed. Sex-specific associations were found between early-life pain and neonatal brain connectivity in female infants only, with greater early-life pain exposure associated with slower maturation in global efficiency (pain × PMA at scan interaction *P* = .002) and local efficiency (pain × PMA at scan interaction *P* = .005). In the full cohort, greater pain exposure was associated with lower global efficiency (coefficient, −0.46; 95% CI, −0.78, to −0.15; *P* = .004) and local efficiency (coefficient, −0.57; 95% CI, −1.04 to −0.10; *P* = .02) and regional connection strength. Local efficiency (coefficient, 0.003; 95% CI, 0.001-0.004; *P* = .005) and regional connection strength in the striatum were associated with cognitive outcomes.

**Conclusions and Relevance:**

In this cohort study of very preterm infants, greater exposure to early-life pain was associated with altered maturation of neonatal structural connectivity, particularly in female infants. Alterations in structural connectivity were associated with neurodevelopmental outcomes, with potential regional specificities.

## Key Points

**Question:**

Are there sex-specific associations among early-life pain exposure, neonatal brain network maturation, and neurodevelopmental outcomes in preterm infants?

**Findings:**

In this prospective cohort study of 150 preterm infants, sex-specific associations were found between early-life pain exposure and maturation of neonatal brain structural connectivity, with a greater association with pain seen in female infants. Decreased brain structural connectivity was associated with poorer 18-month neurodevelopmental outcomes.

**Meaning:**

In this study, early-life pain exposure was associated with slower maturation of structural brain networks, particularly in female infants; clinical trials of neonatal analgesic strategies should consider this sex-specific vulnerability to early-life pain.

## Teaser

This cohort study examines whether greater early-life pain is associated with slower maturation of neonatal connectivity in female vs male infants and whether reduced connectivity is associated with poorer neurodevelopmental outcomes.

## Introduction

Very preterm infants undergo numerous painful procedures in the initial weeks after birth as part of life-saving care.^1^ This care overlaps with a period of rapid brain maturation, including the formation of long- and short-distance structural connections, which may be vulnerable to environmental influences, such as pain exposure.^2,3,4,5^ Greater early-life pain has been associated with altered brain maturation and poorer neurodevelopmental outcomes in children born preterm.^6,7,8,9,10,11,12^ Higher stress exposure, including to invasive procedures, has been associated with slower development of structural connectivity in preterm infants, and lower connectivity in the hippocampus and amygdala has been associated with behavioral outcomes.^13^ Additional studies of the effects of pain on neonatal brain maturation are warranted.

Sex-specific differences in neurodevelopment and brain maturation in preterm infants have been described with more immature neonatal brain maturation and poorer outcomes in male infants, although these differences may not persist long term.^14,15,16,17^ There may also be sex-specific differences in the vulnerability of preterm infants to pain. In animal studies, sexually dimorphic immunologic responses to painful stimuli have been observed, and female rodents were more vulnerable to long-term consequences of early-life pain.^18,19^ In a study of term infants exposed to routine intramuscular injections, female infants demonstrated greater behavioral responses to pain compared with male infants.^20^ In an independent cohort of very preterm infants, we previously observed slower growth of thalamic, basal ganglia, and total brain volumes in female infants exposed to more invasive procedures when compared with male infants.^21^ In the current study, we sought to further characterize the associations among early-life pain, neonatal structural connectivity, and neurodevelopment and explore whether these associations differed by sex. This study is important because there is considerable variability in pain management in preterm infants, likely related to conflicting evidence regarding the efficacy of commonly used analgesic medications.^22,23,24^ Understanding these associations may point toward more effective treatments for pain, which may differ for male and female infants.

In a prospective cohort of very preterm infants who underwent neonatal neuroimaging in the initial weeks after birth and again at term-equivalent age (TEA), we assessed whether there were sex-specific associations between early-life pain and maturation of neonatal structural connectivity and associations of connectivity with 18-month neurodevelopment. We hypothesized that greater early-life pain would be associated with slower maturation of neonatal connectivity in female but not male infants and that reduced connectivity would be associated with poorer neurodevelopmental outcomes.

## Methods

### Study Population

A total of 193 preterm infants (<32 weeks’ gestational age) were recruited prospectively from April 1, 2015, to April 1, 2019, at The Hospital for Sick Children and Mount Sinai Hospital in Toronto, Ontario, Canada. Infants with clinical evidence of congenital infection or genetic syndrome were excluded. This study was approved by research ethics boards at the Hospital for Sick Children and Mount Sinai Hospital. Written informed consent was obtained from a parent or guardian. This cohort study followed the Strengthening the Reporting of Observational Studies in Epidemiology (STROBE) reporting guideline.

### Clinical Data Collection

Given previous findings that high early-life painful exposures are most associated with altered brain maturation, we focused on this period.^10^ We defined early life as the period from birth to early-life magnetic resonance imaging (MRI) (median [IQR] postmenstrual age [PMA], 32.9 [31.7-34.3] weeks) or to a PMA of 32.9 weeks for infants without an early-life MRI. Pain was quantified as the number of invasive procedures, with each attempt counted (eMethods in Supplement 1).^6,10,12^ Definitions of other clinical variables are provided in the eMethods in Supplement 1. Participant ethnicity data were not available at the time points included in this study but will be considered for further follow-up visits.

### Magnetic Resonance Imaging

A total of 186 infants underwent an early-life (n = 173; median [IQR] PMA, 32.9 [31.7-34.3] weeks) and/or TEA (n = 164; median [IQR] PMA, 41.4 [39.4-44.5] weeks) MRI. All infants underwent MRI at 1 site (The Hospital for Sick Children) in an incubator without sedation using a single-channel neonatal head coil, with scanning parameters described in the eMethods in Supplement 1.

#### Brain Injury

Infant MRIs were reviewed by an experienced neuroradiologist (H.M.B.) to identify brain injury. White matter injuries (WMIs) were segmented on 3-dimensional T1-weighted images by a pediatric neurologist (T.S.) using Display software, version 2.0 (McGill University).^25^ Moderate-severe WMIs were defined as injuries with a volume greater than 40 mm^3^.^25^

#### Network Analysis

Details of diffusion tensor imaging preprocessing, tractography, and network analysis are provided in the eMethods in Supplement 1. To identify associations between early-life pain and structural brain network topology, we computed graph theory metrics for each participant, which included the following: (1) global efficiency as a measure of network integration referring to the brain’s ability to integrate specialized information from various brain regions; (2) local efficiency as a measure of network segregation referring to the ability for specialized processing within interconnected brain regions; and (3) small worldness, which assesses for high network segregation and integration, simultaneously allowing for specialized processing and integration of specialized information.^26^ Regional connection strength was computed for each node by determining connections to all other nodes included in the template. We examined thalamocortical, corticostriatal, and thalamostriatal connection strength given previous work showing associations between alterations in these pathways and neurodevelopment^27,28^ and with early-life pain.^10,29^

### Neurodevelopmental Outcomes

Eighteen-month neurodevelopmental assessments were completed with the Bayley Scales of Infant and Toddler Development, Third Edition cognitive, motor, and language composite scores (mean [SD] score, 100 [15]) by an experienced occupational therapist or physiotherapist. Scores ranged from 45 to 155, with scores less than 85 indicating neurodevelopmental concerns.

### Statistical Analysis

Statistical analyses were performed using Stata, version 15.1 (StataCorp LLC) with data analyzed from January 1, 2022, to December 31, 2023. Clinical data were compared between male and female infants using Kruskal-Wallis and Fisher exact tests for continuous and categorical data, respectively.

#### Early-Life Pain and Network Connectivity

Stratifying infants by sex, generalized estimating equations (GEEs) with repeated measures were used to assess whether early-life pain modified maturation of neonatal network connectivity using an interaction term of early-life pain × PMA at scan (3-way interaction of sex × pain × PMA at scan). Univariable GEEs of variables hypothesized to be associated with network topology were performed to identify potential confounders to include in the final regression model. All regressions included PMA at scan and mean fractional anisotropy as covariates. Given that we had 3 measures of network topology, a 2-tailed *P* < .03 was considered significant for interaction terms and *P* < .02 was otherwise considered significant using Bonferroni correction. We assessed associations with thalamocortical, thalamostriatal, and corticostriatal connectivity in similar models; a 2-tailed *P* < .03 was considered significant for interaction terms and *P* < .02 was otherwise considered significant using Bonferroni correction. Similar models were used to assess whether early-life pain modified maturation of regional brain connectivity across all 92 nodes. False discovery rate correction for multiple comparisons was performed using the Simes procedure and a *q* < .05.

#### Neonatal Network Connectivity With Neurodevelopmental Outcomes

We used GEEs to examine associations of whole-brain network connectivity with neurodevelopment while adjusting for PMA at scan, with extreme prematurity on the basis of previous work showing that extreme preterm infants are particularly vulnerable to early-life pain^10^ and with maternal educational level as a marker of socioeconomic status. Additional univariable GEEs of variables hypothesized to be associated with neurodevelopment were performed to identify potential confounders to include in the final regression model. Given that we had 3 measures of whole-brain network connectivity, *P* < .02 was considered significant. We assessed associations of thalamocortical, corticostriatal, and thalamostriatal connectivity with neurodevelopment in similar models; *P* < .02 was considered significant. Similar models were used to assess associations of regional connectivity across all 92 nodes with neurodevelopment while correcting for multiple comparisons using false discovery rate. All GEEs were performed using identity link functions with independent correlation structure and robust SE estimation.

## Results

There were 150 infants (80 [53%] male and 70 [47%] female; median [IQR] gestational age at birth, 27.1 [25.4-29.0] weeks) with structural connectivity data at early life and/or TEA (eFigure 1 in Supplement 1); clinical characteristics by infant sex are given in Table 1. There were differences in birth weight between male and female infants.^30^ Seventy female infants (121 scans) and 80 male infants (133 scans) were included. eTable 1 in Supplement 1 presents clinical characteristics for infants who were included and excluded. With increasing PMA at scan, there was a nonlinear increase in global efficiency, local efficiency, and mean fractional anisotropy but not small worldness (eFigure 2 in Supplement 1).

**Table 1.** Clinical Characteristics of Infants in This Study by Sex^a^

Clinical characteristic	Female infants (n = 70)	Male infants (n = 80)
Birth GA, median (IQR), wk	27 (25.9-28.9)	27.7 (25-29.4)
Extremely preterm infants	44 (63)	43 (54)
Birth weight, median (IQR), g	789 (670-1170)	965 (765-1295)
SGA	9 (13)	8 (10)
Antenatal steroids	56 (80)	67 (84)
Maternal magnesium sulfate exposure	46 (66)	61 (76)
Need for resuscitation at birth	70 (100)	79 (99)
Apgar score at 5 min, median (IQR)	8 (6-9)	8 (7-9)
Hypotension	20 (29)	33 (41)
Culture-positive infections	25 (36)	25 (31)
PDA requiring treatment	23 (33)	18 (23)
ROP requiring treatment	2 (3)	8 (10)
Mechanical ventilation	50 (71)	66 (83)
Days of mechanical ventilation, median (IQR), d	5 (0-20)	4 (1-24)
Chronic lung disease	35 (50)	35 (44)
NEC stage 2 or higher	6 (9)	10 (13)
Major surgery	6 (9)	14 (18)
Moderate-severe WMIs (volume >40 mm^3^)	3 (4)	7 (9)
Intraventricular hemorrhage		
Grade 1	13 (19)	8 (10)
Grade 2	23 (33)	22 (28)
Grade 3	0	1 (1)
Grade 4	0	2 (3)
Early-life invasive procedures, median (IQR)	182 (98-410)	170 (89-462)
Early-life morphine exposure		
None	55 (79)	55 (69)
Short duration	7 (10)	14 (18)
Long duration	8 (11)	11 (14)
Early-life fentanyl exposure		
None	25 (36)	23 (29)
Short duration	38 (54)	43 (54)
Long duration	7 (10)	14 (17)
Early-life midazolam exposure^b^		
None	58	68
Short duration	2 (3)	2 (3)
Postnatal steroids	7 (10)	13 (16)
Maternal educational level^c^		
High school	17 (26)	10 (13)
Undergraduate	44 (68)	56 (73)
Postgraduate	4 (6)	11 (14)

Abbreviations: GA, gestational age; NEC, necrotizing enterocolitis; PDA, patent ductus arteriosus; ROP, retinopathy of prematurity; SGA, small for gestational age; WMIs, white matter injuries.

^a^ Data are presented as number (percentage) of patients unless otherwise indicated.

^b^ Data available for 130 patients.

^c^ Data available in 142 patients.

### Early-Life Pain and Structural Connectivity

#### Structural Brain Network Topology

**Figure 1.**
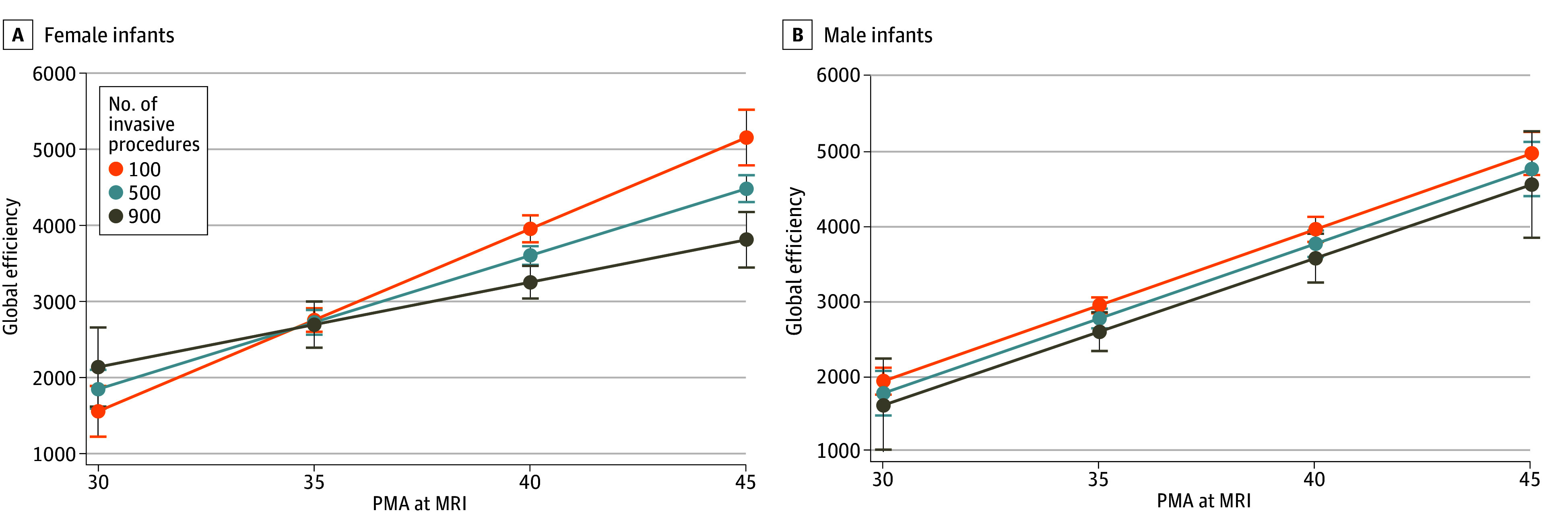
Early-Life Pain × Postmenstrual Age (PMA) at Magnetic Resonance Imaging (MRI) Interaction, Stratified by Sex A. Estimated values of global efficiency with 95% CIs (error bars) for a given number of invasive procedures and PMA at scan showing slower increase in global efficiency by PMA with greater exposure to early-life invasive procedures in female infants (*P* = .002 for interaction). B. Estimated values of global efficiency with 95% CIs for a given number of invasive procedures and PMA at scan showing no significant early-life pain × PMA interaction in male infants (*P* = .90). However, greater exposure to invasive procedures was associated with reduced global efficiency in male infants.

Stratifying by sex, we examined the interaction between early-life pain and PMA at scan with measures of network topology, adjusting for moderate-severe WMIs, which were included as a covariate after univariable analyses (eTable 2 in Supplement 1). In female infants, early-life pain by PMA at scan interaction was significantly associated with global efficiency (pain × PMA at scan interaction *P* = .002) and local efficiency (pain × PMA at scan interaction *P* = .005) but not small worldness (Table 2). Specifically, greater pain exposure was associated with slower maturation of network connectivity with increasing PMA in female infants (Figure 1). However, in male infants, interaction of early-life pain by PMA at scan was not significantly associated with any measure of network topology (Table 2 and Figure 1). In the full cohort, greater early-life pain was associated with lower global (coefficient, −0.46; 95% CI, −0.78 to −0.14; *P* = .004) and local (coefficient, −0.57; 95% CI, −1.04 to −0.10; *P* = .02) efficiency. Additional analyses with the interaction term removed are given in the eResults in Supplement 1. Sensitivity analyses after excluding infants with moderate-severe WMIs are given in the eResults and eTable 3 in Supplement 1, with similar results.

**Table 2.** Generalized Estimating Equations of Early-Life Invasive Procedures × PMA at Scan Interaction and Measures of Network Segregation and Integration Stratified by Sex

Graph measure	Female infants		Male infants	
	Coefficient (95% CI)	*P* value	Coefficient (95% CI)	*P* value
**Global efficiency**				
Early-life pain × PMA at scan	1.0 [Reference]	.002^a^	1.0 [Reference]	.90
Early-life pain	5.55 (1.59 to 9.52)		−0.17 (−4.58 to 4.24)	
PMA at scan	256.56 (203.79 to 309.33)		203.83 (168.66 to 239.00)	
Mean FA	54 179 (45 341.49 to 63 016.51)	<.001^a^	47 858.44 (41 905.41 to 53 811.47)	<.001^a^
Moderate-severe WMIs	−270.98 (−600.00 to 58.03)	.11	−37.14 (−434.56 to 360.28)	.86
**Local efficiency**				
Early-life pain × PMA at scan	1.0 [Reference]	.005^a^	1.0 [Reference]	.86
Early-life pain	6.38 (1.36 to 11.39)		−0.08 (−5.45 to 5.29)	
PMA at scan	377.13 (305.67 to 448.59)		309.92 (263.06 to 356.78)	
Mean FA	65 374.97 (53 897.88 to 76 852.05)	<.001^a^	54 995.15 (46 627.60 to 63 360.70)	<.001^a^
Moderate-severe WMIs	−446.36 (−925.89 to 33.18)	.07	−81.79 (−660.65 to 497.06)	.78
**Small worldness**				
Early-life pain × PMA at scan	1.0 [Reference]	.20	1.0 [Reference]	.23
Early-life pain	0.17 (−0.10 to 0.44)		0.20 (−0.12 to 0.53)	
PMA at scan	9.48 (6.54 to 12.42)		10.48 (7.43 to 13.52)	
Mean FA	−1596.10 (−2250.55 to −941.66)	<.001^a^	−1919.31 (−2283.48 to −1555.13)	<.001^a^
Moderate-severe WMIs	−22.13 (−90.79 to 46.53)	.53	12.03 (−36.78 to 60.84)	.63

Abbreviations: FA, fractional anisotropy; PMA, postmenstrual age; WMIs, white matter injuries.

^a^ *P* < .03 for interaction term and *P* < .02 otherwise.

#### Regional Connectivity

In GEE models stratified by sex, the interaction of early-life pain by PMA at scan was significantly associated with corticostriatal connection in female (pain × PMA at scan interaction *P* = .004) but not male (pain × PMA at scan interaction *P* = .34) infants, adjusting for PMA at scan, mean fractional anisotropy, and moderate-severe WMIs (eTable 4 in Supplement 1). The interaction of early-life pain by PMA at scan was not associated with thalamocortical or thalamostriatal connection strength in female infants, male infants, or the full cohort. For the full cohort, when the interaction was removed, greater early-life pain was associated with lower thalamocortical (coefficient, −0.003; 95% CI, −0.006 to −0.001; *P* = .008) and thalamostriatal (coefficient, −1.17; 95% CI, −1.77 to −0.57; *P* < .001) connection strength.

**Figure 2.**
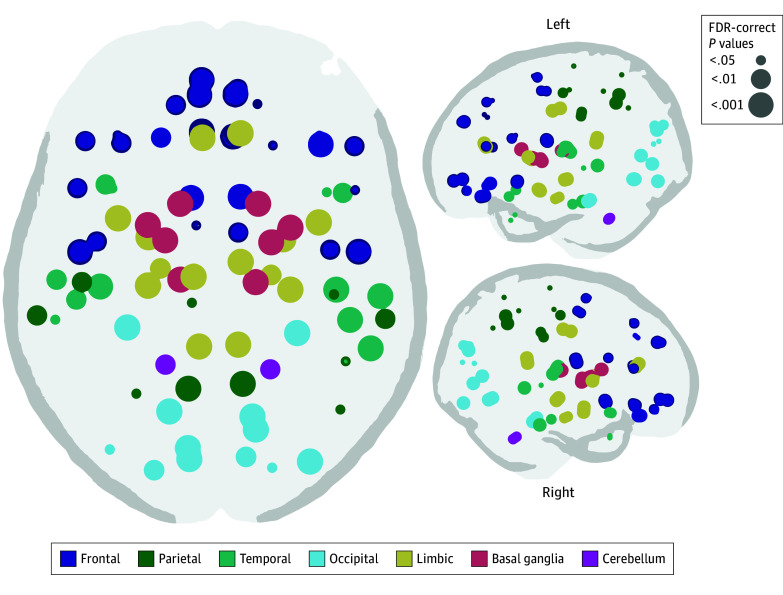
Significant Associations of Early-Life Invasive Procedures With Regional Connectivity Strength Circle size indicates level of significance after correction for multiple comparisons. FDR indicates false discovery rate.

The interaction of early-life pain with PMA at MRI was not significantly associated with connection strength in any of the 92 nodes in the full cohort or when infants were stratified by sex, adjusting for PMA at scan and moderate-severe WMIs. The early-life pain × PMA at MRI interaction term was removed from regression models to assess associations in the full cohort. Greater early-life pain was associated with lower connection strength across several nodes, adjusting for moderate-severe WMIs and correcting for multiple comparisons (Figure 2; eTable 5 in Supplement 1). The strongest associations between pain and regional connection strength were in the basal ganglia, limbic system, motor cortex, and primary visual areas. Associations between early-life pain and structural connectivity remained significant even after accounting for twin pairs.

### Structural Connectivity and Neurodevelopmental Outcomes

A total of 123 infants were seen for neurodevelopmental follow-up. The proportion of children with a history of chronic lung disease differed between those who were and were not seen for follow-up (eTable 6 in Supplement 1). Cognitive, motor, and language outcomes were available for 123, 117, and 119 infants, respectively.

#### Structural Brain Network Topology

In GEE models with neurodevelopmental outcomes as dependent variables, structural brain network topology measures as independent variables, and PMA at scan, mean fractional anisotropy, extreme prematurity, and maternal educational level as covariates, greater local efficiency and small worldness were associated with higher cognitive scores (Table 3). Graph measures of network connectivity were not associated with motor or language outcomes. Maternal educational level was independently associated with cognitive and language outcomes in these models. Additional GEEs were adjusted for clinical factors that were significantly associated with neurodevelopmental outcomes in univariate models (eTable 7 in Supplement 1). After including birth fractional anisotropy, extreme prematurity, days of mechanical ventilatory support, culture-positive infections, and major surgery, greater local efficiency remained significantly associated with higher cognitive scores (eTable 8 in Supplement 1).

**Table 3.** Generalized Estimating Equations of Associations of Measures of Network Segregation and Integration With Bayley Scales of Infant and Toddler Development, Third Edition Scores at 18 Months, Adjusting for PMA at Scan, Extreme Prematurity, and Maternal Educational Level

	Cognitive scores		Motor scores		Language scores	
	Coefficient (95% CI)	*P* value	Coefficient (95% CI)	*P* value	Coefficient (95% CI)	*P* value
**Global efficiency**						
Global efficiency	0.002 (−0.0001 to 0.005)	.05	0.001 (−0.001 to 0.003)	.31	0.001 (−0.002 to 0.004)	.25
PMA at scan	−0.81 (−1.41 to −0.22)	.01^a^	−0.49 (−1.02 to 0.04)	.07	−0.31 (−1.09 to 0.47)	.44
Global FA	−55.22 (−227.68 to 117.25)	.53	−9.10 (−161.17 to 142.98)	.91	12.72 (−194.56 to 220.01)	.90
Extreme prematurity	−3.63 (−8.68 to 1.43)	.16	−4.21 (−8.73 to 0.31)	.07	−6.28 (−12.39 to −0.17)	.04
Maternal educational level						
High school	1.0 [Reference]	NA	1.0 [Reference]	NA	1.0 [Reference]	NA
Undergraduate	6.97 (−0.16 to 13.78)	.05	5.98 (−1.12 to 13.07)	.10	9.39 (1.45 to 17.34)	.02
Postgraduate	17.86 (7.43 to 28.29)	.001^a^	8.21 (0.21 to 16.21)	.04	22.86 (11.96 to 33.76)	<.001^a^
**Local efficiency**						
Local efficiency	0.003 (0.001 to 0.004)	.005^a^	0.001 (−0.001 to 0.002)	.33	0.001 (−0.0001 to 0.004)	.25
PMA at scan	−1.11 (−1.78 to −0.45)	.001^a^	−0.53 (−1.16 to 0.09)	.09	−0.57 (−1.41 to 0.28)	.19
Global FA	−88.47 (−250.73 to 73.79)	.29	−5.27 (−152.3 to 141.76)	.94	−28.71 (−222.71 to 165.28)	.77
Extreme prematurity	−3.42 (−8.42 to 1.57)	.18	−4.22 (−8.72 to 0.29)	.07	−6.06 (−12.17 to 0.04)	.05
Maternal educational level						
High school	1.0 [Reference]	NA	1.0 [Reference]	NA	1.0 [Reference]	NA
Undergraduate	6.97 (0.26 to 13.68)	.04	5.96 (−1.16 to 13.08)	.10	9.42 (1.53 to 17.31)	.02
Postgraduate	17.89 (7.62 to 28.16)	.001^a^	8.19 (0.19 to 16.18)	.05	22.93 (12.1 to 33.76)	<.001^a^
**Small worldness**						
Small worldness	0.03 (0.01 to 0.06)	.01^a^	0.01 (−0.01 to 0.04)	.25	0.03 (−0.003 to 0.06)	.08
PMA at scan	−0.59 (−0.92 to −0.25)	.001^a^	−0.36 (−0.66 to −0.05)	.02	−0.36 (−0.72 to 0.004)	.05
Global FA	−128.36 (43.98 to 212.74)	.003^a^	83.26 (−4.63 to 171.16)	.06	105.48 (−7.53 to 218.5)	.07
Extreme prematurity	−4.56 (−9.63 to 0.51)	.08	−4.42 (−8.98 to 0.13)	.06	−6.95 (−12.88 to −1.03)	.02
Maternal educational level						
High school	1.0 [Reference]	NA	1.0 [Reference]	NA	1.0 [Reference]	NA
Undergraduate	7.26 (0.45 to 14.07)	.04	6.12 (−1.01 to 13.25)	.09	9.67 (1.80 to 17.54)	.02
Postgraduate	18.03 (7.88 to 28.19)	.001^a^	8.30 (0.26 to 16.35)	.04	23.10 (12.44 to 33.77)	<.001^a^

Abbreviations: FA, fractional anisotropy; NA, not applicable; PMA, postmenstrual age.

^a^ *P* < .02.

#### Regional Connectivity

Greater corticostriatal connection strength was associated with higher cognitive (coefficient, 0.08; 95% CI, 0.02-0.15; *P* = .02) and language (coefficient, 0.16; 95% CI, 0.07-0.25; *P* = .001) scores, adjusting for PMA at scan, extreme prematurity, and maternal educational level. Greater thalamostriatal connection strength was associated with higher cognitive scores (coefficient, 0.002; 95% CI, 0.0004-0.004; *P* = .01). There were no associations between thalamocortical connection strength and neurodevelopmental outcomes. The GEEs of regional network connectivity and neurodevelopmental outcomes are given in the eResults and eTable 9 in Supplement 1. Associations between structural connectivity and neurodevelopmental outcomes remained significant even after accounting for twin pairs.

## Discussion

In a prospective cohort of very preterm infants who underwent early-life and TEA brain MRI, we examined sex-specific associations between early-life pain, maturation of neonatal brain structural connectivity, and 18-month neurodevelopment. We found that early-life pain modified maturation of neonatal structural connectivity in female infants, with slower increases in global and local efficiency, and in corticostriatal connectivity in those exposed to greater early-life pain. This interaction was not found in male infants, although greater pain exposure was associated with decreased global and local efficiency and corticostriatal connectivity. In the full cohort, greater pain was also associated with decreased regional connection strength. Collectively, these findings suggest that greater early-life pain is associated with hypoconnectivity in the neonatal brain, with the strongest associations observed in basal ganglia and limbic structures, whereas sex-specific associations suggest that female infants may be more vulnerable to pain. When assessing associations with neurodevelopment, higher global and local efficiency were associated with higher cognitive performance, with potential regional specificity.

The second and third trimesters of gestation are important for the formation and organization of cerebral connectivity, including the development of thalamocortical pathways, growth of long-distance corticocortical fibers, and then short-distance corticocortical connections.^2,3^ Increases in structural connectivity have been observed with increasing PMA in preterm infants,^31,32,33^ including increases in global and local efficiency, measures of network integration, and segregation, which we observed in our cohort. Infants born very preterm have reduced connectivity at TEA when compared with term-born infants, although they have similar overall network architecture. Small-world networks, a key topological feature of the adult connectome that reflects the presence of high network integration and segregation to support efficient information transfer, are present in preterm infants despite overall decreased connectivity.^32,34^ This finding suggests that despite reduced structural connectivity, the preterm brain reorganizes to maximize efficient information flow and transfer, potentially due to the preservation of core connections at the expense of local, short-distance corticocortical connections.^33,35^ This finding could explain why despite overall reduced connectivity with greater painful exposures, there were no associations with small worldness that may have been preserved.

Nociceptive pathways are immature in preterm infants and undergo significant maturational changes in the initial weeks after birth.^36,37^ Repetitive painful exposures can contribute to persistent hyperalgesia and altered neuroendocrine stress responses, which were associated with altered brain maturation and poorer neurodevelopmental outcomes.^36,38,39^ Greater early-life painful exposures have been associated with reduced regional brain volumes, more immature white matter microstructure, and altered functional connectivity.^6,7,10,12,29^ There may be sex-specific differences in vulnerability to long-term adverse effects of pain.^18,19^ In an independent cohort of very preterm infants, slower increases of thalamic, basal ganglia, and total brain volumes were seen in female infants with greater painful exposures than male infants.^21^ Consistent with these prior observations, we observed that greater pain was associated with slower maturation of neonatal connectivity at a whole-brain level and in corticostriatal connection strength in female infants, although greater pain was associated with reduced connectivity in male infants. These sex-specific associations may be mediated by sexually dimorphic hormonal and immunologic responses to pain observed in animal studies,^18,19^ although this warrants further study in preterm infants.

In the full cohort, greater early-life pain was associated with reduced regional connection strength, or diffuse hypoconnectivity. The strongest associations between greater pain and nodal connection strength were in primary motor and visual areas, the first pathways to develop,^2^ and basal ganglia and limbic structures. Associations with connection strength in basal ganglia and limbic structures may be consistent with findings of previous studies. In an independent cohort of preterm infants, early-life pain was associated with smaller thalamic volumes and microstructural alterations in thalamocortical pathways.^10^ Slower growth of neonatal structural connectivity in a large subnetwork involving the limbic system was observed in preterm infants exposed to greater stress during the neonatal intensive care period.^13^ Associations of early pain with altered functional connectivity between the thalamus and somatosensory cortex and in the limbic system have been observed.^21,29^ Collectively, these findings highlight a vulnerability of the basal ganglia and limbic system to early-life pain and stress in preterm infants. Currently, there is a lack of standardized neonatal pain management strategies due to conflicting evidence regarding efficacy and potential harms associated with common analgesic medications.^22,23,40^ Understanding how analgesic medications modify brain networks in a sex-specific manner may provide further insight into optimal pain management strategies. These studies should consider sex-specific effects of early-life pain on neonatal brain maturation and neurodevelopment.

Neonatal alterations in measures of network segregation and integration were associated with lower cognitive scores at 18 months, consistent with previous studies.^27,41,42,43^ In this study, neonatal connection strength within pathways involving the striatum was associated with cognitive and language outcomes. Few studies have examined associations between corticostriatal pathways and neurodevelopment in preterm infants. In school-age children born preterm, altered corticostriatal connectivity was associated with poorer motor outcomes.^28^ Very preterm children were also observed to have slower basal ganglia growth from birth to 7 years of age compared with children born at term, and basal ganglia volumes were associated with poorer neurodevelopmental outcomes.^44^ We did not find significant associations between thalamocortical connection strength and neurodevelopment, which contrasts with previous studies in which weaker thalamocortical connectivity and smaller thalamic volumes were associated with poorer neurodevelopmental outcomes in children born preterm.^27,28,29,45,46^ We hypothesize that associations between the thalamus and neurodevelopment may be mediated through pathways involving the striatum. The role of the striatum in neurodevelopment warrants further investigation in clinical populations of preterm infants and animal models.

### Limitations

This study has some limitations. It is possible that the diffuse alterations in structural connectivity observed in this study are related to increased illness severity as infants with a more complicated neonatal course undergo more invasive procedures. Clinical comorbidities, such as WMIs, have been linked to more immature white matter microstructure and altered neonatal connectivity.^34,47,48,49^ However, even after excluding patients with severe parenchymal brain injury and adjusting for moderate-severe WMI, early-life pain was associated with altered structural connectivity. We also did not observe any associations between clinical illness and neonatal connectivity in our cohort. Regardless, it would be important to explore these associations in larger multicenter cohorts. Birth weight significantly differed between male and female infants with no differences in gestational age at birth, which could be in keeping with known sex-specific differences because the proportion of small-for-gestational-age infants did not differ between groups.^30^ Due to the observational study design, we are unable to make conclusions regarding causation, although the link between early-life pain and neonatal brain development has been previously shown in an animal model.^50^ Findings of altered brain connectivity in relation to early-life pain, with a specific vulnerability of the limbic system and basal ganglia, have now been observed across multiple cohorts at different centers.^10,13,29^ Finally, it would be important to assess relationships with long-term outcomes; longitudinal follow-up is ongoing in this cohort.

## Conclusions

We found sex-specific associations of early-life pain and maturation of neonatal connectivity, with greater painful exposures associated with slower maturation of connectivity in female but not male infants, although greater pain exposure was associated with reduced connectivity in all infants. These findings highlight the importance of minimizing and adequately treating pain in preterm infants. Clinical trials of analgesic medications and pain protocols should consider sex-specific effects on the brain. Finally, altered brain connectivity was associated with neurodevelopment, with a specificity of the striatum. Additional studies with long-term outcomes and focused on understanding the role of the striatum in neurodevelopment are warranted.

## Notes

Supplement 1. **eMethods.** Supplemental Methods **eResults.** Supplemental Results **eFigure 1.** Patient Flowchart **eFigure 2.** Scatterplots of Measures of Network Connectivity and Mean FA by Postmenstrual Age at Scan **eTable 1.** Clinical Characteristics Presented for Infants With and Without Structural Connectivity Data **eTable 2.** Univariable Generalized Estimating Equations of Associations Between Early-Life Painful Exposures and Global Efficiency **eTable 3.** Sensitivity Analyses With Generalized Estimating Equations of Early-Life Invasive Procedures × PMA at Scan Interaction and Measures of Network Segregation and Integration Stratified by Sex After Excluding Patients With Moderate-Severe WMI **eTable 4.** Generalized Estimating Equations of Early-Life Invasive Procedures × Postmenstrual Age at Scan Interaction and Corticostriatal, Thalamocortical and Thalamostriatal Connection Strength, Stratifying by Sex **eTable 5.** Generalized Estimating Equations of Early-Life Invasive Procedures and Regional Connectivity **eTable 6.** Clinical Characteristics for Infants Who Did and Did Not Complete Neurodevelopmental Assessments **eTable 7.** Univariable Generalized Estimating Equations of Associations Between Clinical Variables and Bayley-3 Scores **eTable 8.** Generalized Estimating Equations of Associations of Measures of Network Segregation and Integration With Bayley-3 Scores at 18 Months, Adjusting for Postmenstrual Age at Scan, Global Fractional Anisotropy, Extreme Prematurity, Maternal Education, and Clinical Confounders **eTable 9.** Generalized Estimating Equations of Associations of Regional Connectivity With Bayley-3 Scores at 18 Months, Adjusting for Postmenstrual Age at Scan, Extreme Prematurity, and Maternal Education
eReferences

Supplement 2.
Data Sharing Statement

## References

[1] Carbajal R, Rousset A, Danan C, . Epidemiology and treatment of painful procedures in neonates in intensive care units. JAMA. 2008;300(1):60-70. doi:10.1001/jama.300.1.60 18594041

[2] Kostović I, Radoš M, Kostović-Srzentić M, Krsnik Ž. Fundamentals of the development of connectivity in the human fetal brain in late gestation: from 24 weeks gestational age to term. J Neuropathol Exp Neurol. 2021;80(5):393-414. doi:10.1093/jnen/nlab024 33823016 PMC8054138

[3] Kostović I, Jovanov-Milosević N. The development of cerebral connections during the first 20-45 weeks’ gestation. Semin Fetal Neonatal Med. 2006;11(6):415-422. doi:10.1016/j.siny.2006.07.001 16962836

[4] DeMaster D, Bick J, Johnson U, Montroy JJ, Landry S, Duncan AF. Nurturing the preterm infant brain: leveraging neuroplasticity to improve neurobehavioral outcomes. Pediatr Res. 2019;85(2):166-175. doi:10.1038/s41390-018-0203-9 30531968

[5] Ismail FY, Fatemi A, Johnston MV. Cerebral plasticity: windows of opportunity in the developing brain. Eur J Paediatr Neurol. 2017;21(1):23-48. doi:10.1016/j.ejpn.2016.07.007 27567276

[6] Vinall J, Miller SP, Bjornson BH, . Invasive procedures in preterm children: brain and cognitive development at school age. Pediatrics. 2014;133(3):412-421. doi:10.1542/peds.2013-1863 24534406 PMC3934331

[7] Smith GC, Gutovich J, Smyser C, . Neonatal intensive care unit stress is associated with brain development in preterm infants. Ann Neurol. 2011;70(4):541-549. doi:10.1002/ana.22545 21976396 PMC4627473

[8] Ranger M, Chau CMY, Garg A, . Neonatal pain-related stress predicts cortical thickness at age 7 years in children born very preterm. PLoS One. 2013;8(10):e76702. doi:10.1371/journal.pone.0076702 24204657 PMC3800011

[9] Ranger M, Grunau RE. Early repetitive pain in preterm infants in relation to the developing brain. Pain Manag. 2014;4(1):57-67. doi:10.2217/pmt.13.61 24641344 PMC3975052

[10] Duerden EG, Grunau RE, Guo T, . Early procedural pain is associated with regionally-specific alterations in thalamic development in preterm neonates. J Neurosci. 2018;38(4):878-886. doi:10.1523/JNEUROSCI.0867-17.2017 29255007 PMC5783966

[11] Duerden EG, Grunau RE, Chau V, . Association of early skin breaks and neonatal thalamic maturation: a modifiable risk? Neurology. 2020;95(24):e3420-e3427. doi:10.1212/WNL.0000000000010953 33087497 PMC7836658

[12] Brummelte S, Grunau RE, Chau V, . Procedural pain and brain development in premature newborns. Ann Neurol. 2012;71(3):385-396. doi:10.1002/ana.22267 22374882 PMC3760843

[13] Lammertink F, Benders MJNL, Hermans EJ, . Vulnerability of the neonatal connectome following postnatal stress. J Neurosci. 2022;42(48):8948-8959. doi:10.1523/JNEUROSCI.0176-22.2022 36376077 PMC9732827

[14] Hintz SR, Kendrick DE, Vohr BR, Kenneth Poole W, Higgins RD; Nichd Neonatal Research Network. Gender differences in neurodevelopmental outcomes among extremely preterm, extremely-low-birthweight infants. Acta Paediatr. 2006;95(10):1239-1248. doi:10.1080/08035250600599727 16982497

[15] Nath N, Beltrano W, Haynes L, Dewey D, Bray S. Long-term effects of preterm birth on children’s brain structure: an analysis of the Adolescent Brain Cognitive Development (ABCD) Study. eNeuro. Published online June 5, 2023. doi:10.1523/ENEURO.0196-22.2023 37277147 PMC10262676

[16] Skiöld B, Alexandrou G, Padilla N, Blennow M, Vollmer B, Adén U. Sex differences in outcome and associations with neonatal brain morphology in extremely preterm children. J Pediatr. 2014;164(5):1012-1018. doi:10.1016/j.jpeds.2013.12.051 24530122

[17] Schmidbauer VU, Yildirim MS, Dovjak GO, . Different from the beginning: WM maturity of female and male extremely preterm neonates-a quantitative MRI study. AJNR Am J Neuroradiol. 2022;43(4):611-619. doi:10.3174/ajnr.A7472 35332014 PMC8993206

[18] LaPrairie JL, Murphy AZ. Female rats are more vulnerable to the long-term consequences of neonatal inflammatory injury. Pain. 2007;132(suppl 1):S124-S133. doi:10.1016/j.pain.2007.08.010 17904745 PMC2121098

[19] Sorge RE, Mapplebeck JC, Rosen S, . Different immune cells mediate mechanical pain hypersensitivity in male and female mice. Nat Neurosci. 2015;18(8):1081-1083. doi:10.1038/nn.4053 26120961 PMC4772157

[20] Bellieni CV, Aloisi AM, Ceccarelli D, . Intramuscular injections in newborns: analgesic treatment and sex-linked response. J Matern Fetal Neonatal Med. 2013;26(4):419-422. doi:10.3109/14767058.2012.733777 23039698

[21] Schneider J, Duerden EG, Guo T, . Procedural pain and oral glucose in preterm neonates: brain development and sex-specific effects. Pain. 2018;159(3):515-525. doi:10.1097/j.pain.0000000000001123 29200180

[22] Selvanathan T, Zaki P, McLean MA, . Early-life exposure to analgesia and 18-month neurodevelopmental outcomes in very preterm infants. Pediatr Res. 2023;94(2):738-746. doi:10.1038/s41390-023-02536-y 36859445

[23] Puia-Dumitrescu M, Comstock BA, Li S, ; PENUT Consortium. Assessment of 2-year neurodevelopmental outcomes in extremely preterm infants receiving opioids and benzodiazepines. JAMA Netw Open. 2021;4(7):e2115998. doi:10.1001/jamanetworkopen.2021.15998 34232302 PMC8264640

[24] Borenstein-Levin L, Synnes A, Grunau RE, Miller SP, Yoon EW, Shah PS; Canadian Neonatal Network Investigators. Narcotics and sedative use in preterm neonates. J Pediatr. 2017;180:92-98.e1. doi:10.1016/j.jpeds.2016.08.031 27614931

[25] Cayam-Rand D, Guo T, Synnes A, . Interaction between preterm white matter injury and childhood thalamic growth. Ann Neurol. 2021;90(4):584-594. doi:10.1002/ana.26201 34436793

[26] Rubinov M, Sporns O. Complex network measures of brain connectivity: uses and interpretations. Neuroimage. 2010;52(3):1059-1069. doi:10.1016/j.neuroimage.2009.10.003 19819337

[27] Ball G, Pazderova L, Chew A, . Thalamocortical connectivity predicts cognition in children born preterm. Cereb Cortex. 2015;25(11):4310-4318. doi:10.1093/cercor/bhu331 25596587 PMC4816783

[28] Thompson DK, Loh WY, Connelly A, . Basal ganglia and thalamic tract connectivity in very preterm and full-term children; associations with 7-year neurodevelopment. Pediatr Res. 2020;87(1):48-56. doi:10.1038/s41390-019-0546-x 31486778

[29] Tortora D, Severino M, Di Biase C, . Early pain exposure influences functional brain connectivity in very preterm neonates. Front Neurosci. 2019;13:899. doi:10.3389/fnins.2019.00899 31507370 PMC6716476

[30] Fenton TR, Kim JH. A systematic review and meta-analysis to revise the Fenton growth chart for preterm infants. BMC Pediatr. 2013;13:59. doi:10.1186/1471-2431-13-59 23601190 PMC3637477

[31] Brown CJ, Miller SP, Booth BG, . Structural network analysis of brain development in young preterm neonates. Neuroimage. 2014;101:667-680. doi:10.1016/j.neuroimage.2014.07.030 25076107

[32] van den Heuvel MP, Kersbergen KJ, de Reus MA, . The neonatal connectome during preterm brain development. Cereb Cortex. 2015;25(9):3000-3013. doi:10.1093/cercor/bhu095 24833018 PMC4537441

[33] Batalle D, Hughes EJ, Zhang H, . Early development of structural networks and the impact of prematurity on brain connectivity. Neuroimage. 2017;149:379-392. doi:10.1016/j.neuroimage.2017.01.065 28153637 PMC5387181

[34] Lee JY, Park HK, Lee HJ. Accelerated small-world property of structural brain networks in preterm infants at term-equivalent age. Neonatology. 2019;115(2):99-107. doi:10.1159/000493087 30384384

[35] Ball G, Aljabar P, Zebari S, . Rich-club organization of the newborn human brain. Proc Natl Acad Sci U S A. 2014;111(20):7456-7461. doi:10.1073/pnas.1324118111 24799693 PMC4034228

[36] Fitzgerald M, Walker SM. Infant pain management: a developmental neurobiological approach. Nat Clin Pract Neurol. 2009;5(1):35-50. doi:10.1038/ncpneuro0984 19129789

[37] Fitzgerald M. The development of nociceptive circuits. Nat Rev Neurosci. 2005;6(7):507-520. doi:10.1038/nrn1701 15995722

[38] Brummelte S, Chau CMY, Cepeda IL, . Cortisol levels in former preterm children at school age are predicted by neonatal procedural pain-related stress. Psychoneuroendocrinology. 2015;51:151-163. doi:10.1016/j.psyneuen.2014.09.018 25313535 PMC4268136

[39] Grunau RE, Haley DW, Whitfield MF, Weinberg J, Yu W, Thiessen P. Altered basal cortisol levels at 3, 6, 8 and 18 months in infants born at extremely low gestational age. J Pediatr. 2007;150(2):151-156. doi:10.1016/j.jpeds.2006.10.053 17236892 PMC1851896

[40] McPherson C, Grunau RE. Pharmacologic analgesia and sedation in neonates. Clin Perinatol. 2022;49(1):243-265. doi:10.1016/j.clp.2021.11.014 35210004

[41] Thompson DK, Chen J, Beare R, . Structural connectivity relates to perinatal factors and functional impairment at 7 years in children born very preterm. Neuroimage. 2016;134:328-337. doi:10.1016/j.neuroimage.2016.03.070 27046108 PMC4912891

[42] Fischi-Gómez E, Vasung L, Meskaldji DE, . Structural brain connectivity in school-age preterm infants provides evidence for impaired networks relevant for higher order cognitive skills and social cognition. Cereb Cortex. 2015;25(9):2793-2805. doi:10.1093/cercor/bhu073 24794920

[43] Young JM, Vandewouw MM, Mossad SI, . White matter microstructural differences identified using multi-shell diffusion imaging in six-year-old children born very preterm. Neuroimage Clin. 2019;23:101855. doi:10.1016/j.nicl.2019.101855 31103872 PMC6737393

[44] Loh WY, Anderson PJ, Cheong JLY, . Longitudinal growth of the basal ganglia and thalamus in very preterm children. Brain Imaging Behav. 2020;14(4):998-1011. doi:10.1007/s11682-019-00057-z 30868404

[45] Young JM, Powell TL, Morgan BR, . Deep grey matter growth predicts neurodevelopmental outcomes in very preterm children. Neuroimage. 2015;111:360-368. doi:10.1016/j.neuroimage.2015.02.030 25711136

[46] Loh WY, Anderson PJ, Cheong JLY, . Neonatal basal ganglia and thalamic volumes: very preterm birth and 7-year neurodevelopmental outcomes. Pediatr Res. 2017;82(6):970-978. doi:10.1038/pr.2017.161 28700568 PMC5685902

[47] Duerden EG, Halani S, Ng K, . White matter injury predicts disrupted functional connectivity and microstructure in very preterm born neonates. Neuroimage Clin. 2019;21:101596. doi:10.1016/j.nicl.2018.11.006 30458986 PMC6411591

[48] Glass TJA, Chau V, Gardiner J, . Severe retinopathy of prematurity predicts delayed white matter maturation and poorer neurodevelopment. Arch Dis Child Fetal Neonatal Ed. 2017;102(6):F532-F537. doi:10.1136/archdischild-2016-312533 28536205

[49] Guillot M, Guo T, Ufkes S, . Mechanical ventilation duration, brainstem development, and neurodevelopment in children born preterm: a prospective cohort study. J Pediatr. 2020;226:87-95.e3. doi:10.1016/j.jpeds.2020.05.039 32454115

[50] Anand KJS, Garg S, Rovnaghi CR, Narsinghani U, Bhutta AT, Hall RW. Ketamine reduces the cell death following inflammatory pain in newborn rat brain. Pediatr Res. 2007;62(3):283-290. doi:10.1203/PDR.0b013e3180986d2f 17551412

