# Supplementary material for: Pain Exposure and Brain Connectivity in Preterm Infants
Source: JAMA Netw Open. 2024 Mar 15;7(3):e242551. doi: 10.1001/jamanetworkopen.2024.2551 (PMC10943417; doi:10.1001/jamanetworkopen.2024.2551)
Supplement: Supplement 1. — eMethods. Supplemental Methods eResults. Supplemental Results eFigure 1. Patient Flowchart eFigure 2. Scatterplots of Measures of Network Connectivity and Mean FA by Postmenstrual Age at Scan eTable 1. Clinical Characteristics Presented for Infants With and Without Structural Connectivity Data eTable 2. Univariable Generalized Estimating Equations of Associations Between Early-Life Painful Exposures and Global Efficiency eTable 3. Sensitivity Analyses With Generalized Estimating Equations of Early-Life Invasive Procedures × PMA at Scan Interaction and Measures of Network Segregation and Integration Stratified by Sex After Excluding Patients With Moderate-Severe WMI eTable 4. Generalized Estimating Equations of Early-Life Invasive Procedures × Postmenstrual Age at Scan Interaction and Corticostriatal, Thalamocortical and Thalamostriatal Connection Strength, Stratifying by Sex eTable 5. Generalized Estimating Equations of Early-Life Invasive Procedures and Regional Connectivity eTable 6. Clinical Characteristics for Infants Who Did and Did Not Complete Neurodevelopmental Assessments eTable 7. Univariable Generalized Estimating Equations of Associations Between Clinical Variables and Bayley-3 Scores eTable 8. Generalized Estimating Equations of Associations of Measures of Network Segregation and Integration With Bayley-3 Scores at 18 Months, Adjusting for Postmenstrual Age at Scan, Global Fractional Anisotropy, Extreme Prematurity, Maternal Education, and Clinical Confounders eTable 9. Generalized Estimating Equations of Associations of Regional Connectivity With Bayley-3 Scores at 18 Months, Adjusting for Postmenstrual Age at Scan, Extreme Prematurity, and Maternal Education eReferences [file jamanetwopen-e242551-s001.pdf]

# Supplementary Online Content

Selvanathan T, Ufkes S, Guo T, et al. Pain exposure and brain connectivity in preterm infants. *JAMA Netw Open*. 2024;7(3):e242551. doi:10.1001/jamanetworkopen.2024.2551

**eMethods.** Supplemental Methods
**eResults.** Supplemental Results
**eFigure 1.** Patient Flowchart
**eFigure 2.** Scatterplots of Measures of Network Connectivity and Mean FA by Postmenstrual Age at Scan
**eTable 1.** Clinical Characteristics Presented for Infants With and Without Structural Connectivity Data
**eTable 2.** Univariable Generalized Estimating Equations of Associations Between Early-Life Painful Exposures and Global Efficiency
**eTable 3.** Sensitivity Analyses With Generalized Estimating Equations of Early-Life Invasive Procedures × PMA at Scan Interaction and Measures of Network Segregation and Integration Stratified by Sex After Excluding Patients With Moderate-Severe WMI
**eTable 4.** Generalized Estimating Equations of Early-Life Invasive Procedures × Postmenstrual Age at Scan Interaction and Corticostriatal, Thalamocortical and Thalamostriatal Connection Strength, Stratifying by Sex
**eTable 5.** Generalized Estimating Equations of Early-Life Invasive Procedures and Regional Connectivity
**eTable 6.** Clinical Characteristics for Infants Who Did and Did Not Complete Neurodevelopmental Assessments
**eTable 7.** Univariable Generalized Estimating Equations of Associations Between Clinical Variables and Bayley-3 Scores
**eTable 8.** Generalized Estimating Equations of Associations of Measures of Network Segregation and Integration With Bayley-3 Scores at 18 Months, Adjusting for Postmenstrual Age at Scan, Global Fractional Anisotropy, Extreme Prematurity, Maternal Education, and Clinical Confounders
**eTable 9.** Generalized Estimating Equations of Associations of Regional Connectivity With Bayley-3 Scores at 18 Months, Adjusting for Postmenstrual Age at Scan, Extreme Prematurity, and Maternal Education
**eReferences**

This supplementary material has been provided by the authors to give readers additional information about their work.

© 2024 Selvanathan T et al. *JAMA Network Open.*

## eMETHODS

### Invasive procedures

Each attempt at the following procedures was included, consistent with previous work.[1]

| |
|---|
| Umbilical arterial or venous catheter insertion |
| Peripheral arterial line stab |
| Peripheral intravenous line insertion |
| Peripherally inserted central catheter insertion |
| Venous blood draw |
| Heel poke |
| Intramuscular or subcutaneous injection |
| Chest tube insertion |
| Pleural tap |
| Penrose drain insertion |
| Paracentesis |
| Intubation |
| Suprapubic tap |
| Omaya reservoir tap |
| Circumcision |
| PICC dressing change or removal |
| Intravenous infiltration and extravasation |
| Penrose drain removal |
| Ostomy change |
| Endotracheal tube retaping or suction |
| Nasopharygeal or oropharyngeal suction |
| Orogastric tube insertion and removal |
| Nasogastric/Nasojejunal tube insertion and removal |
| Dressing change |
| X-ray |
| Eye exam |
| Invasive swab |
| Chest compressions |
| Ultrasound |

### Definitions of clinical data

Postnatal culture positive infection was defined as any positive blood or cerebrospinal culture, necrotizing enterocolitis (NEC) as stage 2 or higher, retinopathy of prematurity (ROP) as requiring treatment, chronic lung disease (CLD) as requiring supplemental oxygen at 36 weeks' PMA, and major surgeries as requiring laparotomy, thoracotomy, ostomy, extracorporeal membrane oxygenation or surgery involving the central nervous system. Extreme prematurity was defined as birth gestational age :: 28 weeks. Analgesic exposure (morphine, fentanyl, midazolam) was categorized based on duration of exposure as no exposure, exposure for short durations (7 days or less) or long durations (longer than 7 days).

© 2024 Selvanathan T et al. *JAMA Network Open.*

Intraventricular hemorrhage was scored according to the Papile score.

## MRI Acquisition

Before January 2017, infants were scanned on a 3T Siemens Tim Trio Scanner using the following parameters: (1) Axial 3D T1-weighted images were acquired using fast low-angle shot technique (FLASH) with TE = 5.87 ms, TR = 23 ms, flip angle = 19 degrees, and voxel size = 1.0 x 1.0 x 1.0 $mm^3$; (2) DTI were obtained with a single-shot echo-planar imaging (EPI) sequence with TE = 86 ms, TR = 8000 ms, motion-probing gradient in 30 diffusion-encoding directions with diffusion weighting of 700 $s/mm^3$ (b value) and a non-diffusion weighted image (b=0), FOV = 196 mm, voxel size = 2.0 x 2.0 x 2.0 $mm^3$. After January 2017, infants were scanned on a 3T Siemens Prisma Fit scanner using the following parameters: (1) Axial 3D T1-weighted images acquired using FLASH technique with TE = 2.64 ms, TR = 6.0 ms, flip angle = 9 degrees, and voxel size = 1.0 x 1.0 x 1.0 $mm^3$; (2) DTI were obtained with a single-shot EPI sequence with TE = 87 ms, TR = 8000 ms, motion-probing gradient in 60 diffusion-encoding directions with diffusion weighting of 700 $s/mm^3$ (b value) and three non-diffusion weighted images (b=0), FOV = 219 mm, voxel size = 1.0 x 1.0 x 2.0 $mm^3$.

## DTI Preprocessing and tractography

We excluded scans of 29 patients with extensive brain abnormalities including periventricular hemorrhagic infarction, stroke, brain malformations and severe ventriculomegaly which would have impacted tractography. Data from 11 scans were excluded following visual quality control checks; 141 early-life scans and 131 TEA scans were included in subsequent analyses.

Diffusion images were corrected for head motion (including intervolume motion correction, slice-to-volume motion correction)[2], susceptibility[3], and eddy currents using FSL eddy.[4] Diffusion parameters were estimated in each voxel using FSL BEDPOSTX.[5] We performed probabilistic tractography, allowing fractional anisotropy values to influence the termination of tracts, as implemented in FSL PROBTRACKX2.[6,7] FA is influenced by axonal density, packing, orientation, and myelination, and is typically used as a measure of white matter maturation. Mean fractional anisotropy (FA), a measure of overall connectivity strength, was calculated for each scan using a brain mask.

## Network Construction

To define anatomical regions of interest, we warped neonatal atlases [8,9] to each subject's diffusion image using ANTs [10], first from the atlas to each subject’s T2 image, followed by nonlinear transformations from each T2 image to the corresponding diffusion image. Combining these transformations yielded 92 region labels in each

© 2024 Selvanathan T et al. *JAMA Network Open.*

subject's diffusion space excluding primarily white-matter regions, cerebrospinal fluid, and the brainstem; these were manually reviewed for accuracy of transformations.

All measures were computed using the Brain Connectivity Toolbox for Python.[11] To examine changes in functional segregation and integration, we constructed weighted structural networks for each subject. The 92 anatomical regions were used as nodes. To construct edges, we applied a proportional thresholding approach, in which the ratio of actual connections to possible connections is set to a fixed *density* for all subjects. The weights of the edges connecting two nodes were initially set to the number of tractography streamlines connecting pairs of nodes. The weakest edges were then set to zero, denoting non-connections, until the specified density was reached. Because network properties vary with density, we constructed networks over a range of densities from 0.01 to 0.1 in steps of 0.01. At the density of 0.1, at least 90% of subjects had unthresholded network densities greater than the threshold.

To ensure that our findings were not tied to a specific network density, we used an area under the curve (AUC) approach. Network metrics were computed at each density threshold, and the final value used in analysis was the following modified area under the curve:

$$Y_{\mathrm{AUC}} = \frac{1}{d_n - d_1} \sum_{i=1}^{n-1} (Y_i + Y_{i+1}) \cdot \frac{(d_{i+1} - d_i)}{2}$$

Here, $Y_{\mathrm{i}}$ is the metric value at the $i^{\mathrm{th}}$ network density, $d_{\mathrm{i}}$. The sum represents the area under the metric-density curve. With the coefficient on the sum, the expression represents the mean of the metric over the range of densities, approximated using the trapezoidal rule. This definition returns the metric to its scale on a single network, facilitating its interpretation and comparison with values in other networks.

To measure network integration, we computed *global efficiency* for weighted networks. To measure network segregation, we computed the network *local efficiency* by averaging local efficiency across the network. To measure the extent to which networks exhibited the small world property, we calculated *small-worldness*.

Regional connectivity strength was quantified by the average number of tractography streamlines connecting each region to every other region without thresholding. To measure thalamocortical connection strength, we computed the average number of tractography streamlines connecting the left and right thalamus components to all cortical regions. We used analogous procedures to measure cortico-striatal, and thalamo-striatal connection strengths.

© 2024 Selvanathan T et al. *JAMA Network Open.*

**eResults**

Early-life pain and structural connectivity: Structural brain network topology

The pain by PMA at MRI interaction term was removed to assess whether early-life pain was associated with network topology measures in male infants. Greater early-life pain was associated with lower global (Coef. = -0.46, 95% CI -0.78-(-0.15), p=0.004) and local efficiency (Coef. = -0.57, 95% CI -1.04-(-0.1), p=0.02), but not small worldness (Coef. =0.01, 95% CI -0.03-0.06, p=0.51), adjusting for PMA at MRI, mean FA and moderate-severe WMI.

Sensitivity analyses were performed after removing infants with moderate-severe WMI. Early-life pain by PMA at scan interaction remained significantly associated with global (eTable 3; interaction p=0.002) and local (p=0.005) efficiency in females only, adjusting for PMA at scan, mean FA and moderate-severe WMI.

Structural connectivity and neurodevelopmental outcomes: Regional connectivity

In GEE of regional network connectivity and neurodevelopmental outcomes, greater connection strengths in the left and right putamen were associated with higher Cognitive scores, adjusting for PMA at scan, extreme prematurity, and maternal education, and correcting for multiple comparisons (eTable 8). Greater connection strength in the left caudate was also associated with higher Language scores (eTable 8). There were no significant associations between regional connection strength and motor outcomes in any region. Maternal education was associated with neurodevelopment in these models; children born to mothers who completed postgraduate level education had higher Cognitive, Motor and Language scores compared to mothers whose highest level of education was elementary/high school.

© 2024 Selvanathan T et al. *JAMA Network Open.*

## eFIGURES

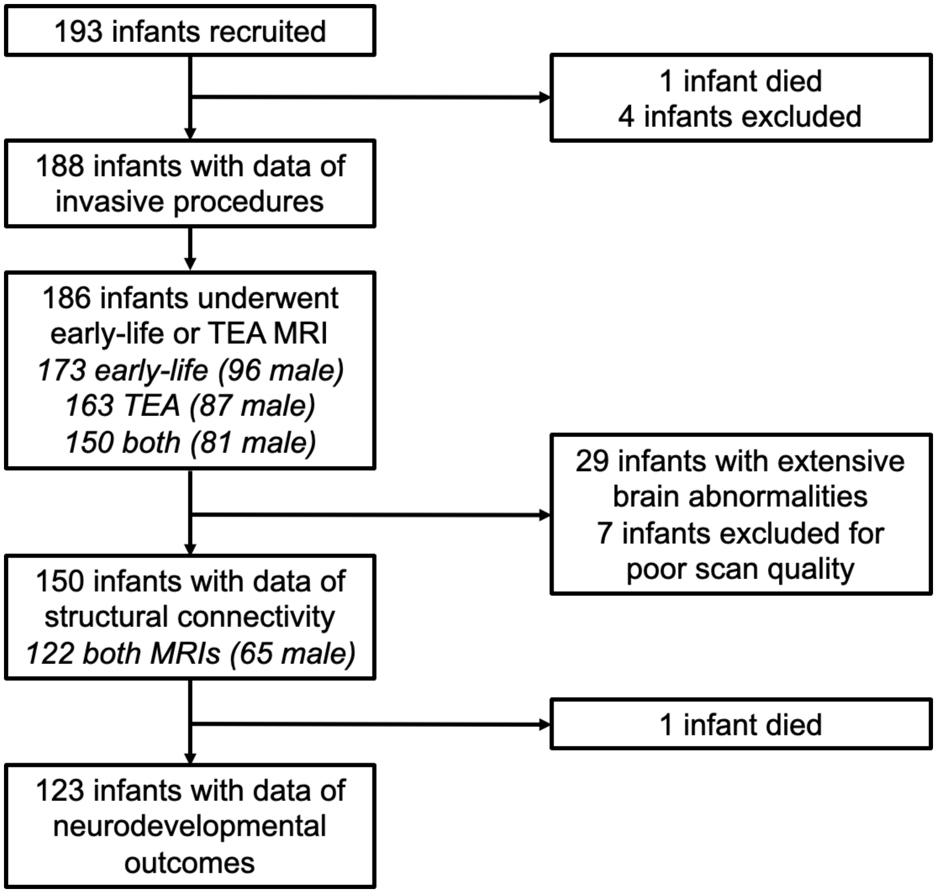


**eFigure 1.** Patient flowchart.

© 2024 Selvanathan T et al. *JAMA Network Open.*

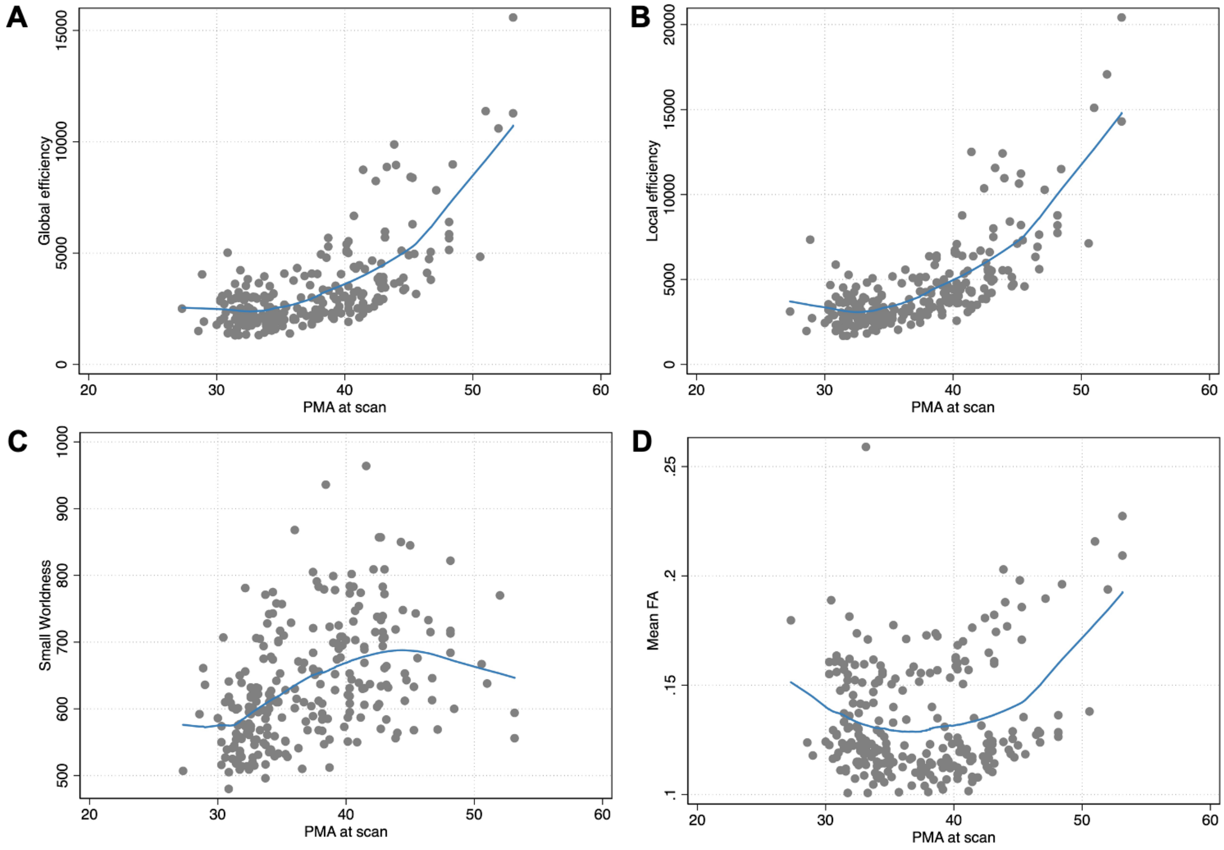


**eFigure 2**. Scatterplots of measures of network connectivity and mean FA by PMA scan. Data from both early-life and term-equivalent age scans included. Fitted line generated using Locally Weighted Scatterplot Smoothing (LOWESS)

Abbreviations: FA = fractional anisotropy; PMA = postmenstrual age

© 2024 Selvanathan T et al. *JAMA Network Open.*

## eTABLES

**eTable 1.** Clinical characteristics presented for infants with and without structural connectivity data.

| Variable | Included (n=150) | Excluded (n=38) | p value |
|---|---|---|---|
| Male | 80 (53) | 24 (63) | 0.36 |
| Birth GA (weeks) | 27.1 (25.4-29.0) | 26.8 (25.0-29.0) | 0.72 |
| Extremely preterm infants | 87 (58) | 22 (58) | 0.57 |
| Birthweight (g) | 930 (700-128=70) | 1000 (750-1195) | 0.68 |
| SGA | 17 (11) | 3 (8) | 0.77 |
| Antenatal steroids | 123 (82) | 30 (79) | 1.0 |
| Maternal magnesium sulphate exposure | 107 (71) | 25 (66) | 0.70 |
| Need for resuscitation at birth | 149 (99) | 38 (100) | 0.80 |
| Apgar at 5 minutes | 8 (6-9) | 6 (4-8) | 0.003* |
| Hypotension | 53 (35) | 14 (37) | 0.56 |
| Culture positive infections | 50 (33) | 14 (37) | 0.70 |
| PDA requiring treatment | 41 (27) | 12 (32) | 0.57 |
| ROP requiring treatment | 10 (7) | 4 (11) | 0.49 |
| Mechanical ventilation | 116 (77) | 32 (84) | 0.03* |
| Days of mechanical ventilation (days) | 4 (1-22) | 10 (2-38) | 0.79 |
| Chronic lung disease | 70 (47) | 12 (32) | 0.04* |
| NEC stage 2 or higher | 16 (11) | 6 (16) | 1.00 |
| Major surgery | 20 (13) | 13 (34) | 0.007* |
| Moderate-severe WMI | 10 (7) | 9 (24) | 0.003* |
| Early-life invasive procedures | 178 (97-426) | 198 (89-502) | 0.72 |
| Early-life morphine exposure[a] | | | 0.04* |
| None | 110 (73) | 21 (57) | |
| Short duration | 21 (14) | 12 (32) | |
| Long duration | 19 (13) | 4 (11) | |
| Maternal education[b] | | | 0.81 |
| High school | 27 (19) | 9 (23) | |
| Undergraduate | 100 (70) | 27 (69) | |
| Postgraduate | 15 (11) | 3 (8) | |

[a]Data available for 187 infants
[b]Data available for 181 infants.

Abbreviations: GA = gestational age; NEC = necrotizing enterocolitis; PDA = patent ductus arteriosus; ROP = retinopathy of prematurity; SGA = small for gestational age; WMI = white matter injury

© 2024 Selvanathan T et al. *JAMA Network Open.*

**eTable 2.** Univariable generalized estimating equations of associations between early-life painful exposures and global efficiency. Similar associations were observed with local efficiency. There were no significant relationships with small worldness.

| Variable | Coef (95% CI) | p value |
|---|---|---|
| Birth gestational age | 75.95 (-8.85-160.76) | 0.08 |
| Extreme prematurity | -410.11 (-895.45-75.23) | 0.10 |
| PDA requiring treatment | -612.41 (-1406.22-181.39) | 0.13 |
| NEC stage 2 or higher | -449.62 (-914.43-15.18) | 0.06 |
| Mechanical ventilation days | 0.16 (-17.96-18.27) | 0.99 |
| Culture positive infections | -78.21 (-569.10-412.68) | 0.76 |
| ROP requiring treatment | 128.75 (-829.92-1087.43) | 0.79 |
| Major surgery | -340.44 (-856.69-175.81) | 0.20 |
| Chronic lung disease | 337.60 (-132.34-807.54) | 0.16 |
| Moderate-severe WMI | 964.71 (44.45-1884.96) | 0.04* |

*p<0.0.05

Abbreviations: CI = confidence interval; Coef = coefficient; PDA = patent ductus arteriosus; NEC = necrotizing enterocolitis; ROP = retinopathy of prematurity; WMI = white matter injury.

© 2024 Selvanathan T et al. *JAMA Network Open.*

**eTable 3.** Sensitivity analyses with generalized estimating equations of early-life invasive procedures x PMA at scan interaction and measures of network segregation and integration stratified by sex after excluding patients with moderate-severe WMI.

| | **Females** | **Males** |
|---|---|---|
| **Network measure** | **Interaction p value** | **Interaction p value** |
| Global efficiency | 0.002 | 0.67 |
| Local efficiency | 0.005 | 0.69 |
| Small worldness | 0.19 | 0.35 |

© 2024 Selvanathan T et al. *JAMA Network Open.*

**eTable 4.** Generalized estimating equations of early-life invasive procedures x postmenstrual age at scan interaction and corticostriatal, thalamocortical and thalamostriatal connection strength, stratifying by sex.

| **Pathway** | **Females** | | **Males** | |
|---|---|---|---|---|
| | **Coef (95% CI)** | **p value** | **Coef (95% CI)** | **p value** |
| **Corticostriatal** | | | | |
| Early-life pain x PMA at scan | - | 0.004* | - | 0.34 |
| Early-life pain | 0.18 (0.05 - 0.3) | | 0.04 (-0.11- 0.19) | |
| PMA at scan | 8.05 (6.2 - 9.91) | | 6.74 (4.88- 8.59) | |
| Moderate-severe WMI | -5.65 (-18.26 - 6.96) | 0.38 | 22.06 (-5.08- 49.19) | 0.11 |
| **Thalamocortical** | | | | |
| Early-life pain x PMA at scan | - | 0.10 | - | 0.40 |
| Early-life pain | -0.03 (-0.05 - 0.001) | | -0.02 (-0.05 - 0.02) | |
| PMA at scan | 0.91 (0.43 - 1.4) | | 1.11 (0.79 - 1.44) | |
| Moderate-severe WMI | -1.46 (-3.41 - 0.49) | 0.14 | -0.83 (-3.82 - 2.16) | 0.59 |
| **Thalamostriatal** | | | | |
| Early-life pain x PMA at scan | - | 0.59 | - | 0.12 |
| Early-life pain | 0.61 (-5.43 - 6.66) | | 4.53 (-2.49- 11.54) | |
| PMA at scan | 371.68 (261.32 - 482.03) | | 446.68 (365.11 - 528.24) | |
| Moderate-severe WMI | 222.17 (-1909.52 -2353.86) | 0.84 | 651.19 (-272.28- 1574.66) | 0.17 |

*p<0.033 for interaction term and <0.017 otherwise

Abbreviations: CI = confidence interval; Coef = coefficient; PMA = postmenstrual age; WMI = white matter injury

© 2024 Selvanathan T et al. *JAMA Network Open.*

**eTable 5.** Generalized estimating equations of early-life invasive procedures and regional connectivity. Q values indicate significance level after FDR correction. Simple model adjusting for postmentstrual age at MRI; complex model adjusting for postmenstrual age at MRI and moderate-severe WMI.

| Region | Simple Model | | | Brain Injury Model | | |
|---|---|---|---|---|---|---|
| | **Coef. 95% CI** | **p** | **q** | **Coef. 95% CI** | **p** | **q** |
| PreCG-L | -0.41 [-0.64-(-0.17)] | 0.001* | 0.002* | -0.38 [-0.61-(-0.15)] | 0.001* | 0.002* |
| PreCG-R | -0.35 [-0.59-(-0.12)] | 0.003* | 0.004* | -0.33 [-0.56-(-0.1)] | 0.005* | 0.007* |
| SFGdor-L | -0.43 [-0.72-(-0.15)] | 0.003* | 0.004* | -0.4 [-0.68-(-0.12)] | 0.005* | 0.007* |
| SFGdor-R | -0.43 [-0.74-(-0.12)] | 0.007* | 0.008* | -0.4 [-0.71-(-0.09)] | 0.01* | 0.01* |
| ORBsup-L | -0.22 [-0.36,(-0.08)] | 0.002* | 0.003* | -0.2 [-0.34-(-0.07)] | 0.003* | 0.004* |
| ORBsup-R | -0.16 [-0.3-(-0.03)] | 0.02* | 0.02* | -0.15 [-0.29-(-0.02)] | 0.03* | 0.03* |
| MFG-L | -0.35 [-0.59-(-0.1)] | 0.005* | 0.006* | -0.33 [-0.57-(-0.08)] | 0.009* | 0.01* |
| MFG-R | -0.31 [-0.57-(-0.04)] | 0.02* | 0.02* | -0.29 [-0.55-(-0.03)] | 0.031* | 0.03* |
| ORBmid-L | -0.07 [-0.16-(0.02)] | 0.11 | 0.11 | -0.06 [-0.15-(-0.02)] | 0.15 | 0.15 |
| ORBmid-R | -0.08 [-0.15-(-0.003)] | 0.04* | 0.04* | -0.07 [-0.14-(0.004)] | 0.06 | 0.06 |
| IFGoperc-L | -0.19 [-0.3-{-0.07)] | 0.001* | 0.002* | -0.18 [-0.29-(-0.07)] | 0.001* | 0.002* |
| IFGoperc-R | -0.21 [-0.35-(-0.06)] | 0.005* | 0.006* | -0.2 [-0.34-(-0.05)] | 0.008* | 0.01* |
| IFGtriang-L | -0.21 [-0.35-(-0.07)] | 0.003* | 0.004* | -0.2 [-0.34-(-0.06)] | 0.005* | 0.007* |
| IFGtriang-R | -0.19 [-0.33-(-0.06)] | 0.004* | 0.005* | -0.19 [-0.32-(-0.05)] | 0.007* | 0.009* |
| ORBinf-L | -0.22 [-0.35-(-0.09)] | 0.001* | 0.002* | -0.2 [-0.33-(-0.08)] | 0.002* | 0.003* |
| ORBinf-R | -0.22 [-0.33-(-0.1)] | <0.001* | <0.001* | -0.2 [-0.32-(-0.09)] | <0.001* | <0.001* |
| ROL-L | -0.26 [-0.38-(-0.13)] | <0.001* | <0.001* | -0.24 [-0.36-(-0.12)] | <0.001* | <0.001* |
| ROL-R | -0.26 [-0.37-(-0.16)] | <0.001* | <0.001* | -0.26 [-0.36-(-0.15)] | <0.001* | <0.001* |
| SMA-L | -0.23 [-0.38-(-0.07)] | 0.005* | 0.006* | -0.2 [-0.35-(-0.05)] | 0.01* | 0.01* |
| SMA-R | -0.3 [-0.48-(-0.11)] | 0.002* | 0.003* | -0.27 [-0.45-(-0.09)] | 0.003* | 0.004* |
| OLF-L | -0.2 [-0.27-(-0.14)] | <0.001* | <0.001* | -0.2 [-0.26-(-0.14)] | <0.001* | <0.001* |
| OLF-R | -0.18 [-0.23-(-0.12)] | <0.001* | <0.001* | -0.17 [-0.22-(-0.12)] | <0.001* | <0.001* |
| SFGmed-L | -0.42 [-0.64-(-0.2)] | <0.001* | <0.001* | -0.39 [-0.61-(-0.17)] | <0.001* | <0.001* |
| SFGmed-R | -0.36 [-0.57-(-0.15)] | 0.001* | 0.002* | -0.34 [-0.54-(-0.13)] | 0.001* | 0.002* |
| ORBmed-L | -0.15 [-0.22-(-0.08)] | <0.001* | <0.001* | -0.14 [-0.21-(-0.07)] | <0.001* | <0.001* |
| ORBmed-R | -0.19 [-0.27-(-0.1)] | <0.001* | <0.001* | -0.18 [-0.26-(-0.1)] | <0.001* | <0.001* |
| REC-L | -0.26 [-0.38-(-0.13)] | <0.001* | <0.001* | -0.24 [-0.36-(-0.12)] | <0.001* | <0.001* |
| REC-R | -0.22 [-0.34-(-0.11)] | <0.001* | <0.001* | -0.21 [-0.32-(-0.1)] | <0.001* | <0.001* |
| INS-L | -0.47 [-0.62-(-0.33)] | <0.001* | <0.001* | -0.46 [-0.6-(-0.32)] | <0.001* | <0.001* |
| INS-R | -0.44 [-0.57-(-0.32)] | <0.001* | <0.001* | -0.43 [-0.55-(-0.31)] | <0.001* | <0.001* |
| ACG-L | -0.4 [-0.53-(-0.28)] | <0.001* | <0.001* | -0.4 [-0.52-(-0.27)] | <0.001* | <0.001* |
| ACG-R | -0.33 [-0.44-(-0.22)] | <0.001* | <0.001* | -0.32 [-0.43-(-0.21)] | <0.001* | <0.001* |
| MCG-L | -0.48 [-0.68-(-0.29)] | <0.001* | <0.001* | -0.46 [-0.65-(-0.27)] | <0.001* | <0.001* |
| MCG-R | -0.58 [-0.81-(-0.35)] | <0.001* | <0.001* | -0.55 [-0.77-(-0.33)] | <0.001* | <0.001* |
| PCG-L | -0.26 [-0.32-(-0.2)] | <0.001* | <0.001* | -0.25 [-0.31-(-0.19)] | <0.001* | <0.001* |
| PCG-R | -0.2 [-0.25-(-0.15)] | <0.001* | <0.001* | -0.2 [-0.25-(-0.15)] | <0.001* | <0.001* |
| HIP-L | -0.15 [-0.23-(-0.07)] | <0.001* | <0.001* | -0.14 [-0.22-(-0.07)] | <0.001* | <0.001* |
| HIP-R | -0.16 [-0.25-(-0.07)] | <0.001* | <0.001* | -0.15 [-0.24-(-0.07)] | <0.001* | <0.001* |
| PHG-L | -0.2 [-0.31-(-0.08)] | 0.001* | 0.002* | -0.19 [-0.3-(-0.08)] | 0.001* | 0.002* |
| PHG-R | -0.24 [-0.38-(-0.1)] | 0.001* | 0.002* | -0.23 [-0.36-(-0.1)] | 0.001* | 0.002* |
| AMYG-L | -0.06 [-0.09-(-0.03)] | <0.001* | <0.001* | 0.06 [-0.09-(-0.03)] | <0.001* | <0.001* |

© 2024 Selvanathan T et al. *JAMA Network Open.*

| | | | | | | |
|---|---|---|---|---|---|---|
| AMYG-R | -0.06 [-0.09-(-0.03)] | <0.001* | <0.001* | -0.06 [-0.09-(-0.03)] | <0.001* | <0.001* |
| CAL-L | -0.62 [-0.87-(-0.36)] | <0.001* | <0.001* | -0.59 [-0.84-(-0.35)] | <0.001* | <0.001* |
| CAL-R | -0.49 [-0.66-(-0.32)] | <0.001* | <0.001* | -0.47 [-0.63-(-0.3)] | <0.001* | <0.001* |
| CUN-L | -0.53 [-0.76-(-0.31)] | <0.001* | <0.001* | -0.51 [-0.73-(-0.29)] | <0.001* | <0.001* |
| CUN-R | -0.48 [-0.75-(-0.21)] | <0.001* | <0.001* | -0.46 [-0.72-(-0.2)] | 0.001* | 0.002* |
| LING-L | -0.51 [-0.72-(-0.3)] | <0.001* | <0.001* | -0.49 [-0.69-(-0.28)] | <0.001* | <0.001* |
| LING-R | -0.63 [-0.84-(-0.41)] | <0.001* | <0.001* | -0.59 [-0.8-(-0.39)] | <0.001* | <0.001* |
| SOG-L | -0.38 [-0.59-(-0.17)] | <0.001* | <0.001* | -0.36 [-0.57-(-0.15)] | 0.001* | 0.002* |
| SOG-R | -0.32 [-0.56-(-0.07)] | 0.01* | 0.01* | -0.3 [-0.54-(-0.06)] | 0.01* | 0.02* |
| MOG-L | -0.32 [-0.55-(-0.08)] | 0.008* | 0.009* | -0.28 [-0.51-(-0.06)] | 0.01* | 0.02* |
| MOG-R | -0.24 9-0.41-(-0.07)] | 0.006* | 0.007* | -0.22 [-0.39-(-0.05)] | 0.009* | 0.01* |
| IOG-L | -0.17 [-0.34-(-0.01)] | 0.06 | 0.06 | -0.14 [-0.31-(0.02)] | 0.09 | 0.09 |
| IOG-R | -0.24 [-0.35-(-0.13)] | <0.001* | <0.001* | -0.21 [-0.31-(-0.11)] | <0.001* | <0.001* |
| FFG-L | -0.5 [-0.77-(-0.24)] | <0.001* | <0.001* | -0.48 [-0.73-(-0.22 | <0.001* | <0.001* |
| FFG-R | -0.66 [-0.95-(-0.38)] | <0.001* | <0.001* | -0.62 [-0.9-(-0.34)] | <0.001* | <0.001* |
| PoCG-L | -0.4 [-0.68-(-0.13)] | 0.004* | 0.005* | -0.37 [-0.64-(-0.1)] | 0.007* | 0.009* |
| PoCG-R | -0.35 [-0.61-(-0.1)] | 0.006* | 0.007* | -0.32 [-0.57-(-0.07)] | 0.01* | 0.02* |
| SPG-L | -0.16 [-0.31-(-0.02)] | 0.03* | 0.03* | -0.15 [-0.3-(-0.01)] | 0.04* | 0.04* |
| SPG-R | -0.15 [-0.33-(-0.02)] | 0.09 | 0.09 | -0.13 [-0.3-(-0.04)] | 0.15 | 0.15 |
| IPL-L | -0.22 [-0.43-(-0.01)] | 0.04* | 0.04* | -0.2 [-0.4-(-0.01)] | 0.06 | 0.06 |
| IPL-R | -0.18 [-0.31-(-0.05)] | 0.006* | 0.007* | -0.15 [-0.26-(-0.04)] | 0.01* | 0.01* |
| SMG-L | -0.2 [-0.34-(-0.06)] | 0.004* | 0.005* | -0.19 [-0.32-(-0.05)] | 0.007* | 0.009* |
| SMG-R | -0.23 [-0.36-(-0.1)] | 0.001* | 0.002* | -0.2 [-0.33-(-0.08)] | 0.002* | 0.003* |
| ANG-L | -0.11 [-0.26-(-0.04)] | 0.14 | 0.14 | -0.09 [-0.24-(-0.05)] | 0.195 | 0.19 |
| ANG-R | -0.19 [-0.33-(-0.05)] | 0.009* | 0.01* | -0.16 [-0.29-(-0.03)] | 0.02* | 0.02* |
| PCUN-L | -0.72 [-1.05-(-0.38)] | <0.001* | <0.001* | -0.677 [-1.01-(-0.35)] | <0.001* | <0.001* |
| PCUN-R | -0.75 [-1,08-(-0.43)] | <0.001* | <0.001* | -0.71 [-103-(-0.4)] | <0.001* | <0.001* |
| PCL-L | -0.14 [-0.24-(-0.03)] | 0.01* | 0.01* | -0.12 [-0.22-(-0.02)] | 0.022* | 0.03* |
| PCL-R | -0.11 [-0.19-(-0.04)] | 0.004* | 0.005* | -0.1 [-0.17-(-0.03)] | 0.009* | 0.01* |
| CAU-L | -0.14 [-0.21-(-0.07)] | <0.001* | <0.001* | -0.14 [-0.2-(-0.07)] | <0.001* | <0.001* |
| CAU-R | -0.15 [-0.23-(-0.08)] | <0.001* | <0.001* | -0.15 [-0.22-(-0.08)] | <0.001* | <0.001* |
| PUT-L | -0.47 [-0.6-(-0.35)] | <0.001* | <0.001* | -0.47 [-0.59-(-0.34)] | <0.001* | <0.001* |
| PUT-R | -0.47 [-0.61-(-0.34)] | <0.001* | <0.001* | -0.46 [-0.6-(-0.33)] | <0.001* | <0.001* |
| PAL-L | -0.27 [-0.37-(-0.17)] | <0.001* | <0.001* | -0.28 [-0.37-(-0.18)] | <0.001* | <0.001* |
| PAL-R | -0.25 [-0.34-(-0.17)] | <0.001* | <0.001* | -0.25 [-0.33-(-0.16)] | <0.001* | <0.001* |
| THA-L | -0.18 [-0.26-(-0.1)] | <0.001* | <0.001* | -0.18 [-0.25-(-0.1)] | <0.001* | <0.001* |
| THA-R | -0.11 [-0.17-(-0.06)] | <0.001* | <0.001* | -0.11 [-0.16-(-0.50)] | <0.001* | <0.001* |
| HES-L | -0.1 [-0.14-(-0.06)] | <0.001* | <0.001* | -0.1 [-0.14-(-0.06)] | <0.001* | <0.001* |
| HES-R | -0.08 [-0.11-(-0.05)] | <0.001* | <0.001* | -0.08 [-0.11-(-0.05)] | <0.001* | <0.001* |
| STG-L | -0.36 [-0.57-(-0.16)] | <0.001* | <0.001* | -0.35 [-0.55-(-0.15)] | 0.001* | 0.002* |
| STG-R | -0.36 [-0.5-(-0.22)] | <0.001* | <0.001* | -0.34 [-0.47-(-0.2)] | <0.001* | <0.001* |
| TPOsup-L | -0.19 [-0.31-(-0.07)] | 0.002* | 0.003* | -0.18 [-0.29-(-0.06)] | 0.004* | 0.006* |
| TPOsup-R | -0.21 [-0.35-(-0.08)] | 0.001* | 0.003* | -0.2 [-0.33-(-0.07)] | 0.003* | 0.004* |
| MTG-L | -0.36 [-0.66-(-0.05)] | 0.02* | 0.02* | -0.33 [-0.63-(-0.03)] | 0.03* | 0.04* |
| MTG-R | -0.4 [-0.6-(-0.21)] | <0.001* | <0.001* | -0.37 [-0.55-(-0.18)] | <0.001* | <0.001* |
| TPOmid-L | -0.1 [-0.19-(-0.02)] | 0.02* | 0.02* | -0.09 [-0.28-(-0.08)] | 0.03* | 0.04* |
| TPOmid-R | -0.14 [-0.24-(-0.04)] | 0.005* | 0.006* | -0.13 [-0.23-(-0.03)] | 0.009* | 0.01* |
| ITG-L | -0.37 [-0.57-(-0.16)] | <0.001* | <0.001* | -0.34 [-0.55-(-0.14)] | 0.001* | 0.002* |
| ITG-R | -0.45 [-0.67-(-0.22)] | <0.001* | <0.001* | -0.41 [-0.63-(-0.19)] | <0.001* | <0.001* |

© 2024 Selvanathan T et al. *JAMA Network Open.*

| | | | | | | |
|---|---|---|---|---|---|---|
| CBH-L | -0.38 [-0.61-(-0.15)] | 0.001* | 0.002* | -0.35 [-0.58-(-0.12)] | 0.003* | 0.004* |
| CBH-R | -0.4 [-0.63-(-0.17)] | 0.001* | 0.002* | -0.37 [-0.6-(-0.14)] | 0.002* | 0.003* |

*p<0.05

Abbreviations: ACG = anterior cingulate gyrus; AMYG = amygdala; ANG = angular gyrus; CAL = calcarine cortex; CAU = caudate; CBH = cerebellar hemisphere; Coef = coefficient; CUN = cuneus; FDR = false discovery rate; FFG = fusiform gyrus; HES = Heschl gyrus; HIP = hippocampus; IFGoperc = inferior frontal gyrus (opercular); IFGtriang = inferior frontal gyrus (triangular); INS = insula; IOG = inferior occipital gyrus; IPL = inferior parietal lobe; ITG = inferior temporal gyrus; L = left; LING = lingual gyrus; MCG = middle cingulate gyrus; MFG = middle frontal gyrus; MOG = middle occipital gyrus; MTG = middle temporal gyrus; OLF = olfactory; ORBinf = orbitofrontal cortex (inferior); ORBmed = orbitofrontal cortex (medial); ORBmid = orbitofrontal cortex (middle); ORBsup = orbitofrontal cortex (superior); PAL = pallidum; PCG = posterior cingulate gyrus; PVL = paracentral lobe; PCUN = precuneus; PHG = parahippocampal gyrus; PreCG = precentral gyrus; PoCG = postcentral gyrus; PUT = putamen; REC = rectus gyrus; ROL = Rolandic cortex; R = right; SFGdor = superior frontal gyrus (dorsal); SFGmed = superior frontal gyrus (medial); SMA = supplementary motor area; SMG = supramarginal gyrus; SOG = superior occipital gyrus; SPG = superior parietal gyrus; STG = superior temporal gyrus; THA = thalamus; TPOsup = temporal pole (superior); TPOmid = temporal pole (middle)

© 2024 Selvanathan T et al. *JAMA Network Open.*

**eTable 6.** Clinical characteristics for infants who did and did not complete neurodevelopmental assessments.

| Clinical characteristic | Infants seen in follow-up (n=123) | Infants not seen in follow-up (n=27) | P value |
|---|---|---|---|
| Male | 68 (55) | 12 (44) | 0.40 |
| Birth GA (weeks) | 27.1 (25.4-28.9) | 27.6 (25-30.6) | 0.48 |
| Extremely preterm infants | 72 (59) | 15 (56) | 0.83 |
| Birthweight (g) | 930 (710-1280) | 920 (670-1230) | 0.80 |
| SGA | 14 (11) | 3 (11) | 1.00 |
| Antenatal steroids | 103 (84) | 20 (74) | 0.27 |
| Maternal magnesium sulphate exposure | 89 (72) | 18 (67) | 0.47 |
| Need for resuscitation at birth | 122 (99) | 27 (100) | 1.00 |
| Apgar at 5 minutes | 8 (6-9) | 8 (5-9) | 0.73 |
| Hypotension | 46 (37) | 7 (26) | 0.37 |
| Culture positive infections | 41 (33) | 9 (33) | 1.00 |
| PDA requiring treatment | 34 (28) | 7 (26) | 1.00 |
| ROP requiring treatment | 8 (7) | 2 (7) | 1.00 |
| Mechanical ventilation | 93 (76) | 23 (85) | 0.32 |
| Days of mechanical ventilation (days) | 5 (1-23) | 3 (1-15) | 0.79 |
| Chronic lung disease | 63 (51) | 7 (26) | 0.02* |
| NEC stage 2 or higher | 13 (11) | 3 (11) | 1.00 |
| Major surgery | 16 (13) | 4 (25) | 0.76 |
| Early-life invasive procedures | 178 (97-435) | 177 (88-368) | 0.60 |
| Early-life morphine exposure | | | 0.74 |
| None | 91 (74) | 19 (70) | |
| Short duration | 16 (13) | 5 (19) | |
| Long duration | 16 (13) | 3 (11) | |
| Maternal education[a] | | | 0.20 |
| High school | 20 (17) | 7 (33) | |
| Undergraduate | 87 (72) | 13 (62) | |
| Postgraduate | 14 (11) | 1 (5) | |
| Died | - | 1 | - |

[a]Data available for 142 infants.
Data presented as No. (%) or median (IQR).

Abbreviations: GA = gestational age; NEC = necrotizing enterocolitis; PDA = patent ductus arteriosus; ROP = retinopathy of prematurity; SGA = small for gestational age

**eTable 7.** Univariable generalized estimating equations of associations between clinical variables and Bayley-3 scores.

| Variable | Cognitive | Motor | Language |
|---|---|---|---|
| Birth gestational age | 1.66 (0.54-2.78) P=0.004* | 1.27 (0.37-2.17) P=0.006* | 2.09 (0.77-3.41) P=0.002* |
| Extreme prematurity | -4.66 (-10.05-0.73) P=0.09 | -3.58 (-8.19-1.03) P=0.13 | -7.29 (-13.54-(-1.04)) P=0.02* |
| PDA requiring treatment | -6.86 (-14.78-1.06) P=0.09 | -2.39 (-8.95-4.16) P=0.47 | -7.46 (-17.02-2.10) P=0.13 |
| NEC stage 2 or higher | -2.25 (-11.21-6.71) P=0.62 | -6.71 (-15.19-1.77) P=0.12 | -7.64 (-18.55-3.26) P=0.17 |
| Mechanical ventilation days | -0.14 (-0.25-(-0.03)) P=0.02* | -0.19 (-0.30-(-0.08)) P=0.001* | -0.22 (-0.37-(-0.07)) P=0.005* |
| Culture positive infections | -5.33 (-10.91-0.26) P=0.06 | -3.97 (-9.36-1.41) P=0.15 | -7.87 (-14.81-(-0.52) P=0.04* |
| ROP requiring treatment | -10.30 (-20.94-0.35) P=0.06 | -9.07 (-19.81-1.67) P=0.10 | -5.19 (-19.74-9.35) P=0.48 |
| Major surgery | -3.16 (-9.51-3.18) P=0.33 | -6.31 (-11.84-0.78) P=0.03* | -10.53 (-19.29-(-1.77)) P=0.02* |
| Chronic lung disease | -1.19 (-6.52-4.15) P=0.66 | -0.83 (-5.49-3.83) P=0.73 | -3.06 (-9.47-3.34) P=0.35 |
| Moderate-severe WMI | 4.41 (-1.45-10.27) P=0.14 | 2.19 (-3.84-8.21) P=0.48 | 7.27 (-2.22-16.75) P=0.13 |
| Maternal Education | | | |
| High school | Ref | Ref | Ref |
| Undergraduate | 5.59 (-0.80-11.97) P=0.09 | 4.05 (-2.25-10.36) P=0.21 | 7.45 (-0.5-15.40) P=0.07 |
| Postgraduate | 15.64 (5.0-26.28) P=0.004* | 7.74 (0.01-15.48) P=0.05 | 21.63 (10.48-32.77) P<0.001* |

*p<0.05

Abbreviations: GA = gestational age; NEC = necrotizing enterocolitis; PDA = patent ductus arteriosus; ROP = retinopathy of prematurity; SGA = small for gestational age

© 2024 Selvanathan T et al. *JAMA Network Open.*

**eTable 8.** Generalized estimating equations of associations of measures of network segregation and integration with Bayley-3 scores at 18 months, adjusting for postmenstrual age at scan, global fractional anisotropy, extreme prematurity, maternal education, and clinical confounders

| **Network measure** | **Cognitive**<br>Coef (95% CI)<br>P value | **Motor**<br>Coef (95% CI)<br>P value | **Language**<br>Coef (95% CI)<br>P value |
|---|---|---|---|
| Global Efficiency | 0.002 (0.0003-0.004)<br>P=0.03 | 0.001 (-0.001-0.003)<br>P=0.37 | 0.001 (-0.002-0.004)<br>P=0.61 |
| Local Efficiency | 0.002 (0.001-0.004)<br>P=0.002 | 0.001 (-0.001-0.002)<br>P=0.34 | 0.001 (-0.001-0.003)<br>P=0.23 |
| Small Worldness | 0.02 (-0.0002-0.05)<br>P=0.05 | 0.01 (-0.01-0.03)<br>P=0.28 | 0.02 (-0.004-0.05)<br>P=0.09 |

*Models adjusting for: birth gestational age, days of mechanical ventilation, culture positive infections, and major surgery.

Abbreviations: CI = confidence interval; Coef = coefficient.

© 2024 Selvanathan T et al. *JAMA Network Open.*

**eTable 9.** Generalized estimating equations of associations of regional connectivity with Bayley-3 scores at 18 months, adjusting for postmenstrual age at scan, extreme prematurity, and maternal education. q value indicates significance after FDR correction.

| Region | Cognitive Scores | | | Motor Scores | | | Language Scores | | |
|---|---|---|---|---|---|---|---|---|---|
| | Coef. 95% CI | p | q | Coef. 95% CI | p | q | Coef 95% CI | p | q |
| PreCG-L | 0.003 (-0.001-0.01) | 0.16 | 0.31 | 0.002 (-0.001-0.01) | 0.16 | 0.45 | 0.002 (-0.001-0.01) | 0.21 | 0.72 |
| PreCG-R | 0.003 (-0.001-0.01) | 0.12 | 0.29 | 0.002 (-0.001-0.01) | 0.17 | 0.45 | 0.002 (-0.001-0.01) | 0.16 | 0.72 |
| SFGdor-L | 0.002 (-0.001-0.004) | 0.25 | 0.41 | 0.002 (-0.0001-0.004) | 0.07 | 0.45 | 0.001 (-0.002-0.004) | 0.54 | 0.75 |
| SFGdor-R | 0.002 (-0.0003-0.005) | 0.09 | 0.28 | 0.002 (-0.001-0.004) | 0.15 | 0.45 | 0.002 (-0.001-0.005) | 0.14 | 0.72 |
| ORBsup-L | 0.006 (-0.0001-0.01) | 0.06 | 0.25 | 0.001 (-0.004-0.01) | 0.75 | 0.84 | 0.01 (-0.003-0.01) | 0.19 | 0.72 |
| ORBsup-R | 0.004 (-0.003-0.01) | 0.27 | 0.41 | 0.0004 (-0.01-0.01) | 0.90 | 0.94 | 0.004 (-0.004-0.01) | 0.32 | 0.72 |
| MFG-L | 0.002 (-0.001-0.005) | 0.25 | 0.41 | 0.002 (-0.001-0.005) | 0.13 | 0.45 | 0.001 (-0.002-0.01) | 0.58 | 0.76 |
| MFG-R | 0.002 (-0.001-0.01) | 0.17 | 0.32 | 0.002 (-0.001-0.004) | 0.19 | 0.45 | 0.002 (-0.002-0.01) | 0.29 | 0.72 |
| ORBmid-L | 0.004 (-0.01-0.01) | 0.48 | 0.57 | -0.0004 (-0.01-0.01) | 0.94 | 0.94 | 0.01 (-0.01-0.02) | 0.34 | 0.72 |
| ORBmid-R | 0.01 (-0.01-0.02) | 0.28 | 0.41 | 0.002 (-0.01-0.01) | 0.67 | 0.80 | 0.01 (-0.01-0.02) | 0.44 | 0.72 |
| IFGoperc-L | 0.01 (-0.001-0.02) | 0.10 | 0.28 | 0.005 (-0.002-0.01) | 0.20 | 0.45 | 0.003 (-0.01-0.01) | 0.49 | 0.72 |
| IFGoperc-R | 0.003 (-0.002-0.01) | 0.25 | 0.41 | 0.004 (-0.002-0.01) | 0.17 | 0.45 | 0.003 (-0.003-0.01) | 0.28 | 0.72 |
| IFGtriang-L | 0.003 (-0.003-0.01) | 0.34 | 0.45 | 0.001 (-0.004-0.01) | 0.60 | 0.73 | 0.002 (-0.01-0.01) | 0.55 | 0.75 |
| IFGtriang-R | 0.002 (-0.004-0.01) | 0.44 | 0.55 | 0.002 (-0.003-0.01) | 0.39 | 0.55 | 0.003 (-0.004-0.01) | 0.42 | 0.72 |
| ORBinf-L | 0.001 (-0.01-0.01) | 0.71 | 0.74 | -0.0005 (-0.01-0.01) | 0.87 | 0.94 | 0.00001 (-0.01-0.01) | 1.00 | 1.00 |
| ORBinf-R | 0.003 (-0.01-0.01) | 0.55 | 0.62 | 0.0005 (-0.01-0.01) | 0.90 | 0.94 | 0.003 (-0.01 ,0.01) | 0.53 | 0.75 |
| ROL-L | 0.005 (-0.0002-0.01) | 0.06 | 0.26 | 0.005 (0.0001-0.01) | 0.04 | 0.45 | 0.004 (-0.002-0.01) | 0.18 | 0.72 |
| ROL-R | 0.004 (-0.001-0.01) | 0.14 | 0.30 | 0.01 (-0.002-0.01) | 0.15 | 0.45 | 0.005 (-0.003-0.01) | 0.25 | 0.72 |
| SMA-L | 0.003 (-0.002-0.01) | 0.22 | 0.38 | 0.002 (-0.002-0.01) | 0.25 | 0.47 | 0.002 (-0.004-0.01) | 0.47 | 0.72 |
| SMA-R | 0.004 (-0.0001-0.01) | 0.06 | 0.25 | 0.002 (-0.002-0.01) | 0.27 | 0.47 | 0.003 (-0.002-0.01) | 0.20 | 0.72 |
| OLF-L | 0.01 (-0.002-0.03) | 0.09 | 0.28 | 0.01 (-0.01-0.02) | 0.26 | 0.47 | 0.02 (-0.001-0.04) | 0.06 | 0.59 |
| OLF-R | 0.02 (-0.003-0.04) | 0.02 | 0.16 | 0.01 (-0.003-0.03) | 0.10 | 0.45 | 0.02 (-0.002-0.04) | 0.03 | 0.59 |
| SFGmed-L | 0.001 (-0.003-0.005) | 0.66 | 0.71 | 0.002 (-0.001-0.005) | 0.27 | 0.47 | 0.001 (-0.004-0.01) | 0.82 | 0.86 |
| SFGmed-R | 0.001 (-0.003-0.01) | 0.54 | 0.61 | 0.002 (-0.002-0.005) | 0.37 | 0.53 | 0.001 (-0.004-0.01) | 0.63 | 0.77 |
| ORBmed-L | 0.01 (-0.01-0.02) | 0.31 | 0.43 | 0.001 (-0.01-0.01) | 0.83 | 0.90 | 0.01 (-0.01-0.02) | 0.40 | 0.72 |
| ORBmed-R | 0.004 | 0.51 | 0.59 | 0.0004 | 0.93 | 0.94 | 0.01 | 0.29 | 0.72 |

© 2024 Selvanathan T et al. *JAMA Network Open.*

| | | | | | | | | | |
|---|---|---|---|---|---|---|---|---|---|
| | (-0.01-0.02) | | | (-0.01-0.01) | | | (-0.01-0.02) | | |
| REC-L | 0.01 (-0.001-0.01) | 0.14 | 0.30 | 0.0004 (-0.01-0.01) | 0.88 | 0.94 | 0.01 (-0.003-0.01) | 0.24 | 0.72 |
| REC-R | 0.004 (-0.004-0.01) | 0.33 | 0.45 | 0.001 (-0.01-0.01) | 0.82 | 0.90 | 0.01 (-0.004-0.01) | 0.29 | 0.72 |
| INS-L | 0.01 (0.002-0.01) | 0.007* | 0.16 | 0.01 (0.001-0.01) | 0.01 | 0.45 | 0.005 (-0.002-0.01) | 0.18 | 0.72 |
| INS-R | 0.004 (-0.001-0.01) | 0.10 | 0.28 | 0.01 (-0.0002-0.01) | 0.06 | 0.45 | 0.01 (-0.0002-0.02) | 0.04* | 0.59 |
| ACG-L | 0.004 (-0.003-0.01) | 0.24 | 0.41 | 0.004 (-0.003-0.01) | 0.23 | 0.46 | 0.005 (-0.005-0.01) | 0.33 | 0.72 |
| ACG-R | 0.01 (-0.001-0.02) | 0.07 | 0.28 | 0.01 (-0.002-0.01) | 0.16 | 0.45 | 0.01 (-0.002-0.02) | 0.10 | 0.72 |
| MCG-L | 0.004 (0.001-0.01) | 0.02* | 0.16 | 0.002 (-0.001-0.01) | 0.17 | 0.45 | 0.003 (-0.002-0.01) | 0.29 | 0.72 |
| MCG-R | 0.004 (0.0005-0.01) | 0.02* | 0.16 | 0.002 (-0.001-0.004) | 0.27 | 0.47 | 0.003 (-0.001-0.01) | 0.18 | 0.72 |
| PCG-L | 0.02 (0.0003-0.03) | 0.05 | 0.24 | 0.01 (-0.001-0.03) | 0.03 | 0.45 | 0.01 (-0.01-0.03) | 0.29 | 0.72 |
| PCG-R | 0.02 (0.0001-0.05) | 0.05 | 0.24 | 0.02 (-0.001-0.04) | 0.04 | 0.45 | 0.02 (-0.01-0.05) | 0.24 | 0.72 |
| HIP-L | 0.002 (-0.01-0.01) | 0.58 | 0.64 | 0.01 (-0.003-0.01) | 0.22 | 0.45 | 0.001 (-0.01-0.01) | 0.76 | 0.81 |
| HIP-R | 0.003 (-0.01-0.01) | 0.48 | 0.57 | 0.002 (-0.01-0.01) | 0.56 | 0.70 | 0.002 (-0.01-0.01) | 0.68 | 0.77 |
| PHG-L | 0.001 (-0.005-0.01) | 0.65 | 0.71 | 0.002 (-0.003-0.01) | 0.41 | 0.58 | 0.001 (-0.004-0.01) | 0.69 | 0.77 |
| PHG-R | 0.002 (-0.004-0.01) | 0.48 | 0.57 | 0.002 (-0.003-0.01) | 0.42 | 0.58 | 0.002 (-0.004-0.01) | 0.48 | 0.72 |
| AMYG-L | 0.004 (-0.02-0.03) | 0.70 | 0.74 | 0.01 (-0.01-0.03) | 0.20 | 0.45 | 0.01 (-0.01-0.02) | 0.62 | 0.77 |
| AMYG-R | -0.001 (-0.02-0.02) | 0.91 | 0.93 | 0.001 (-0.02-0.02) | 0.92 | 0.94 | 0.002 (-0.03-0.02) | 0.86 | 0.88 |
| CAL-L | 0.002 (-0.001-0.005) | 0.14 | 0.30 | 0.001 (-0.001-0.004) | 0.18 | 0.45 | 0.001 (-0.001-0.004) | 0.34 | 0.72 |
| CAL-R | 0.004 (-0.001-0.01) | 0.11 | 0.29 | 0.001 (-0.003-0.005) | 0.70 | 0.82 | 0.001 (-0.004-0.01) | 0.75 | 0.80 |
| CUN-L | 0.004 (0.001-0.01) | 0.02* | 0.16 | 0.002 (-0.001-0.01) | 0.17 | 0.45 | 0.002 (-0.002-0.005) | 0.38 | 0.72 |
| CUN-R | 0.003 (0.0002-0.01) | 0.03* | 0.19 | 0.001 (-0.002-0.003) | 0.50 | 0.66 | 0.001 (-0.002-0.003) | 0.57 | 0.75 |
| LING-L | 0.002 (-0.001-0.01) | 0.20 | 0.37 | 0.002 (-0.001-0.005) | 0.13 | 0.45 | 0.001 (-0.002-0.005) | 0.39 | 0.72 |
| LING-R | 0.003 (-0.0003-0.01) | 0.08 | 0.28 | 0.001 (-0.002-0.004) | 0.41 | 0.58 | 0.002 (-0.003-0.01) | 0.47 | 0.72 |
| SOG-L | 0.003 (0.0004-0.01) | 0.02* | 0.16 | 0.002 (-0.001-0.004) | 0.29 | 0.48 | 0.002 (-0.001-0.01) | 0.21 | 0.72 |
| SOG-R | 0.003 (0.0001-0.01) | 0.04* | 0.21 | 0.001 (-0.002-0.003) | 0.60 | 0.73 | 0.001 (-0.002-0.003) | 0.48 | 0.72 |
| MOG-L | 0.002 (-0.001-0.004) | 0.28 | 0.41 | 0.001 (-0.001-0.003) | 0.30 | 0.48 | 0.001 (-0.002-0.004) | 0.47 | 0.72 |
| MOG-R | 0.004 (0.001-0.01) | 0.01* | 0.16 | 0.002 (-0.002-0.01) | 0.36 | 0.53 | 0.003 (-0.001-0.01) | 0.16 | 0.72 |
| IOG-L | 0.001 (-0.002-0.005) | 0.45 | 0.55 | 0.001 (-0.001-0.003) | 0.23 | 0.46 | 0.001 (-0.002-0.004) | 0.50 | 0.73 |
| IOG-R | 0.01 (-0.001-0.01) | 0.08 | 0.28 | 0.003 (-0.002-0.01) | 0.19 | 0.45 | 0.005 (-0.004-0.01) | 0.29 | 0.72 |
| FFG-L | 0.002 (-0.001-0.005) | 0.26 | 0.41 | 0.001 (-0.001-0.003) | 0.19 | 0.45 | 0.001 (-0.002-0.004) | 0.54 | 0.75 |
| FFG-R | 0.002 (-0.0004-0.004) | 0.11 | 0.28 | 0.001 (-0.001-0.003) | 0.18 | 0.45 | 0.001 (-0.002-0.004) | 0.49 | 0.72 |

© 2024 Selvanathan T et al. *JAMA Network Open.*

| | | | | | | | | | |
|---|---|---|---|---|---|---|---|---|---|
| PoCG-L | 0.002 (-0.0003-0.005) | 0.09 | 0.28 | 0.002 (-0.001-0.004) | 0.12 | 0.45 | 0.002 (-0.001-0.01) | 0.17 | 0.72 |
| PoCG-R | 0.002 (-0.001-0.004) | 0.16 | 0.32 | 0.002 (-0.001-0.004) | 0.30 | 0.48 | 0.002 (-0.001-0.005) | 0.23 | 0.72 |
| SPG-L | 0.004 (-0.001-0.01) | 0.12 | 0.29 | 0.003 (-0.002-0.01) | 0.27 | 0.47 | 0.001 (-0.005-0.01) | 0.63 | 0.77 |
| SPG-R | 0.002 (-0.001-0.01) | 0.14 | 0.30 | 0.002 (-0.002-0.004) | 0.54 | 0.69 | 0.001 (-0.003-0.004) | 0.64 | 0.77 |
| IPL-L | 0.002 (-0.001-0.01) | 0.18 | 0.33 | 0.002 (-0.001-0.005) | 0.19 | 0.45 | 0,001 (-0.003-0.005) | 0.67 | 0.77 |
| IPL-R | 0.001 (-0.002-0.004) | 0.68 | 0.73 | 0.001 (-0.003-0.004) | 0.60 | 0.73 | 0.0004 (-0.004-0.01) | 0.88 | 0.90 |
| SMG-L | 0.004 (-0.001-0.01) | 0.16 | 0.31 | 0.004 (0.00003-0.01) | 0.05 | 0.45 | 0.003 (-0.002-0.01) | 0.27 | 0.72 |
| SMG-R | 0.003 (-0.002-0.01) | 0.25 | 0.41 | 0.002 (-0.004-0.01) | 0.52 | 0.69 | 0.002 (-0.004-0.01) | 0.48 | 0.72 |
| ANG-L | 0.002 (-0.002-0.01) | 0.39 | 0.50 | 0.002 (-0.001-0.005) | 0.17 | 0.45 | 0.001 (-0.003-0.004) | 0.70 | 0.77 |
| ANG-R | 0.002 (-0.002-0.01) | 0.35 | 0.46 | 0.001 (-0.003-0.01) | 0.54 | 0.69 | 0.001 (-0.004-0.01) | 0.75 | 0.80 |
| PCUN-L | 0.002 (0.0001-0.004) | 0.04 | 0.21 | 0.001 (-0.001-0.004) | 0.21 | 0.45 | 0.001 (-0.001-0.004) | 0.36 | 0.72 |
| PCUN-R | 0.002 (0.0004-0.004) | 0.02* | 0.16 | 0.001 (-0.001-0.003) | 0.33 | 0.52 | 0.001 (-0.001-0.003) | 0.38 | 0.72 |
| PCL-L | 0.005 (-0.004-0.01) | 0.29 | 0.41 | 0.004 (-0.004-0.01) | 0.34 | 0.52 | 0.004 (-0.004-0.01) | 0.35 | 0.72 |
| PCL-R | 0.005 (-0.004-0.01) | 0.29 | 0.41 | 0.004 (-0.005-0.01) | 0.34 | 0.52 | 0.002 (-0.01-0.01) | 0.67 | 0.77 |
| CAU-L | 0.01 (-0.003-0.03) | 0.12 | 0.29 | 0.01 (-0.01-0.02) | 0.28 | 0.48 | 0.03 (0.01-0.04) | <0.001* | <0.001* |
| CAU-R | 0.02 (0.005-0.03) | 0.006* | 0.16 | 0.01 (0.001-0.03) | 0.04 | 0.45 | 0.02 (-0.001-0.03) | 0.06 | 0.59 |
| PUT-L | 0.01 (-0.004-0.02) | 0.001* | 0.04* | 0.01 (-0.0004-0.01) | 0.07 | 0.45 | 0.01 (0.004-0.02) | 0.003* | 0.14 |
| PUT-R | 0.01 (0.004-0.02) | 0.001* | 0.04* | 0.01 (0.001-0.01) | 0.03 | 0.45 | 0.01 (0.003-0.02) | 0.008* | 0.18 |
| PAL-L | 0.01 (-0.002-0.02) | 0.11 | 0.28 | 0.004 (-0.01-0.01) | 0.44 | 0.59 | 0.01 (-0.00004-0.03) | 0.05 | 0.59 |
| PAL-R | 0.01 (0.001-0.02) | 0.02 | 0.16 | 0.01 (-0.004-0.01) | 0.25 | 0.47 | 0.01 (-0.001-0.02) | 0.06 | 0.59 |
| THA-L | 0.01 (0.002-0.02) | 0.02 | 0.16 | 0.01 (-0.003-0.02) | 0.14 | 0.45 | 0.01 (-0.004-0.03) | 0.17 | 0.72 |
| THA-R | 0.01 (-0.01-0.02) | 0.31 | 0.43 | 0.003 (-0.01-0.02) | 0.68 | 0.80 | 0.004 (-0.02-0.03) | 0.73 | 0.80 |
| HES-L | 0.01 (-0.01-0.02) | 0.42 | 0.52 | 0.01 (-0.002-0.03) | 0.10 | 0.45 | 0.01 (-0.01-0.04) | 0.35 | 0.72 |
| HES-R | 0.02 (0.004-0.04) | 0.02* | 0.16 | 0.02 (-0.002-0.04) | 0.07 | 0.45 | 0.04 (0.01-0.06) | 0.008* | 0.18 |
| STG-L | 0.002 (-0.002-0.01) | 0.35 | 0.45 | 0.002 (-0.0004-0.01) | 0.10 | 0.45 | 0.002 (-0.002-0.01) | 0.30 | 0.72 |
| STG-R | 0.004 (-0.001-0.01) | 0.14 | 0.30 | 0.004 (-0.001-0.01) | 0.09 | 0.45 | 0.003 (-0.003-0.01) | 0.32 | 0.72 |
| TPOsup-L | -0.0002 (-0.01-0.01) | 0.95 | 0.96 | 0.001 (-0.005-0.01) | 0.75 | 0.84 | -0.002 (-0.01-0.01) | 0.57 | 0.75 |
| TPOsup-R | 0.0002 (-0.01-0.01) | 0.96 | 0.96 | 0.001 (-0.005-0.01) | 0.74 | 0.84 | 0.0004 (-0.01-0.01) | 0.91 | 0.92 |
| MTG-L | 0.001 (-0.001-0.003) | 0.5 | 0.59 | 0.001 (-0.0005-0.003) | 0.16 | 0.45 | 0.001 (-0.001-0.003) | 0.48 | 0.72 |
| MTG-R | 0.003 (-0.001-0.01) | 0.19 | 0.35 | 0.002 (-0.001-0.005) | 0.21 | 0.45 | 0.002 (-0.002-0.007) | 0.33 | 0.72 |

© 2024 Selvanathan T et al. *JAMA Network Open.*

| | | | | | | | | | |
|---|---|---|---|---|---|---|---|---|---|
| TPOmid-L | 0.002<br>(-0.01-0.01) | 0.72 | 0.75 | 0.0004<br>(-0.01-0.01) | 0.93 | 0.94 | -0.003<br>(-0.02-0.01) | 0.68 | 0.77 |
| TPOmid-R | 0.005<br>(-0.004-0.01) | 0.28 | 0.41 | 0.003<br>(-0.003-0.01) | 0.36 | 0.53 | 0.002<br>(-0.007-0.01) | 0.61 | 0.77 |
| ITG-L | 0.002<br>(-0.002-0.01) | 0.29 | 0.41 | 0.002<br>(-0.001-0.005) | 0.15 | 0.45 | 0.001<br>(-0.003-0.01) | 0.55 | 0.75 |
| ITG-R | 0.003<br>(-0.0004-0.01) | 0.10 | 0.28 | 0.001<br>(-0.001-0.004) | 0.21 | 0.45 | 0.002<br>(-0.001-0.01) | 0.25 | 0.72 |
| CBH-L | 0.002<br>(-0.0002-0.01) | 0.07 | 0.28 | 0.002<br>(-0.00003-0.005) | 0.05 | 0.45 | 0.001<br>(-0.002-0.01) | 0.47 | 0.72 |
| CBH-R | 0.002<br>(-0.0004-0.01) | 0.1 | 0.28 | 0.002<br>(0.0001-0.01) | 0.04 | 0.45 | 0.001<br>(-0.003-0.005) | 0.66 | 0.77 |

*p value <0.05
*q value <0.05

Abbreviations: ACG = anterior cingulate gyrus; AMYG = amygdala; ANG = angular gyrus; CAL = calcarine cortex; CAU = caudate; CBH = cerebellar hemisphere; Coef = coefficiency; CUN = cuneus; FDR = false discovery rate; FFG = fusiform gyrus; HES = Heschl gyrus; HIP = hippocampus; IFGoperc = inferior frontal gyrus (opercular); IFGtriang = inferior frontal gyrus (triangular); INS = insula; IOG = inferior occipital gyrus; IPL = inferior parietal lobe; ITG = inferior temporal gyrus; L = left; LING = lingual gyrus; MCG = middle cingulate gyrus; MFG = middle frontal gyrus; MOG = middle occipital gyrus; MTG = middle temporal gyrus; OLF = olfactory; ORBinf = orbitofrontal cortex (inferior); ORBmed = orbitofrontal cortex (medial); ORBmid = orbitofrontal cortex (middle); ORBsup = orbitofrontal cortex (superior); PAL = pallidum; PCG = posterior cingulate gyrus; PVL = paracentral lobe; PCUN = precuneus; PHG = parahippocampal gyrus; PreCG = precentral gyrus; PoCG = postcentral gyrus; PUT = putamen; REC = rectus gyrus; ROL = Rolandic cortex; R = right; SFGdor = superior frontal gyrus (dorsal); SFGmed = superior frontal gyrus (medial); SMA = supplementary motor area; SMG = supramarginal gyrus; SOG = superior occipital gyrus; SPG = superior parietal gyrus; STG = superior temporal gyrus; THA = thalamus; TPOsup = temporal pole (superior); TPOmid = temporal pole (middle)

© 2024 Selvanathan T et al. *JAMA Network Open.*

## eReferences


1. Vinall J, Miller SP, Bjornson BH, et al. Invasive procedures in preterm children: brain and cognitive development at school age. *Pediatrics*. 2014;133(3):412-21. doi:10.1542/peds.2013-1863
2. Andersson JLR, Graham MS, Drobnjak I, Zhang H, Filippini N, Bastiani M. Towards a comprehensive framework for movement and distortion correction of diffusion MR images: Within volume movement. *Neuroimage*. May 15 2017;152:450-466. doi:10.1016/j.neuroimage.2017.02.085
3. Andersson JLR, Graham MS, Drobnjak I, Zhang H, Campbell J. Susceptibility-induced distortion that varies due to motion: Correction in diffusion MR without acquiring additional data. *Neuroimage*. May 1 2018;171:277-295. doi:10.1016/j.neuroimage.2017.12.040
4. Andersson JLR, Sotiropoulos SN. An integrated approach to correction for off-resonance effects and subject movement in diffusion MR imaging. *Neuroimage*. Jan 15 2016;125:1063-1078. doi:10.1016/j.neuroimage.2015.10.019
5. Jbabdi S, Sotiropoulos SN, Savio AM, Graña M, Behrens TEJ. Model-based analysis of multishell diffusion MR data for tractography: How to get over fitting problems. *Magnetic Resonance in Medicine*. 2012-12-01 2012;68(6):1846-1855. doi:10.1002/mrm.24204
6. Behrens TEJ, Berg HJ, Jbabdi S, Rushworth MFS, Woolrich MW. Probabilistic diffusion tractography with multiple fibre orientations: What can we gain? *NeuroImage*. 2007-01-01 2007;34(1):144-155. doi:10.1016/j.neuroimage.2006.09.018
7. Behrens TEJ, Woolrich MW, Jenkinson M, et al. Characterization and propagation of uncertainty in diffusion-weighted MR imaging. *Magnetic Resonance in Medicine*. 2003-11-01 2003;50(5):1077-1088. doi:10.1002/mrm.10609
8. Oishi K, Mori S, Donohue PK, et al. Multi-contrast human neonatal brain atlas: Application to normal neonate development analysis. *NeuroImage*. 2011-05-01 2011;56(1):8-20. doi:10.1016/j.neuroimage.2011.01.051
9. Shi F, Yap P-T, Wu G, et al. Infant Brain Atlases from Neonates to 1- and 2-Year-Olds. *PLoS ONE*. 2011-04-14 2011;6(4):e18746. doi:10.1371/journal.pone.0018746
10. Tustison NJ, Cook PA, Klein A, et al. Large-scale evaluation of ANTs and FreeSurfer cortical thickness measurements. *Neuroimage*. Oct 1 2014;99:166-79. doi:10.1016/j.neuroimage.2014.05.044
11. Rubinov M, Sporns O. Complex network measures of brain connectivity: Uses and interpretations. *NeuroImage*. 2010-09-01 2010;52(3):1059-1069. doi:10.1016/j.neuroimage.2009.10.003


© 2024 Selvanathan T et al. *JAMA Network Open.*
