# Supplementary material for: Pain Exposure and Brain Connectivity in Preterm Infants
Source: JAMA Netw Open. 2024 Mar 15;7(3):e242551. doi: 10.1001/jamanetworkopen.2024.2551 (PMC10943417; doi:10.1001/jamanetworkopen.2024.2551)
Supplement: Supplement 2. — Data Sharing Statement [file jamanetwopen-e242551-s002.pdf]

Data Sharing Statement

Selvanathan. Pain Exposure and Brain Connectivity in Preterm Infants. *JAMA Netw Open*. Published March 15, 2024. doi:10.1001/jamanetworkopen.2024.2551

Data
Data available: No

Additional Information
Explanation for why data not available: Data can be made available upon reasonable request and with appropriate Research Ethics Boards and Data Sharing Agreements in place.
